# Comparison of Efficacy and Safety of Single and Double Immune Checkpoint Inhibitor-Based First-Line Treatments for Advanced Driver-Gene Wild-Type Non-Small Cell Lung Cancer: A Systematic Review and Network Meta-Analysis

**DOI:** 10.3389/fimmu.2021.731546

**Published:** 2021-08-16

**Authors:** Qian Xu, Xue Zhang, Miao Huang, Xin Dai, Jing Gao, Song Li, Lei Sheng, Kai Huang, Jian Wang, Lian Liu

**Affiliations:** ^1^Department of Medical Oncology, Qilu Hospital, Cheeloo College of Medicine, Shandong University, Jinan, China; ^2^Department of Medical Oncology, Shandong Provincial Hospital of Traditional Chinese Medicine, Jinan, China; ^3^Department of Thyroid Surgery, General Surgery, Qilu Hospital, Cheeloo College of Medicine, Shandong University, Jinan, China

**Keywords:** non-small cell lung cancer, first-line, immune checkpoint inhibitors, single, double, network meta-analysis

## Abstract

**Background:**

Immune checkpoint inhibitors (ICIs) have improved survival for advanced wild-type non-small cell lung cancer (NSCLC) significantly, but few studies compared single ICI (SICI)-based treatments and double ICIs (DICI)-based treatments. We summarized the general efficacy of ICI-related treatments, compared the efficacy and safety of SICI-based [programmed death 1 (PD-1)/programmed death-ligand 1 (PD-L1) or cytotoxic T lymphocyte-associated antigen 4 (CTLA-4) inhibitors ± chemotherapy (CT)] and DICI-based (PD-1/PD-L1 inhibitors+CTLA-4 inhibitors ± chemotherapy) treatments *vs*. CT in the first-line treatment.

**Methods:**

We included phase II/III randomized controlled trials (RCTs), including patients with histologically confirmed stage IIIB–IV driver-gene wild-type NSCLC who received first-line ICI-related therapy in at least one arm. PubMed, Embase, and Cochrane Library were searched from January 1, 2005, to December 31, 2020. This network meta-analysis was performed in a Bayesian framework using GEMTC and JAGS package in R.3.6.1. The research was registered with PROSPERO (CRD42020184534).

**Results:**

Twenty RCTs were involved, including 13,032 patients and 17 treatment regimens. The results showed that ICI-based therapies could provide a pooled median overall survival (mOS) (POS) of 15.79 (95% CI: 14.85–16.73) months, and there were no significant differences in OS, progression-free survival (PFS), objective response rate (ORR), and grade 3 or higher adverse events (≥3AEs) between DICI-based treatments (POS: 14.81, 12.11–17.52 months) and SICI-based treatments (POS: 16.17, 14.59–17.74 months) in overall patients. However, DICI-based treatments had significantly prolonged the OS over SICI-based treatments in squamous and PD-L1 <1% subgroups. The ranking of OS benefit by Bayesian surface under the cumulative ranking curve (SUCRA) spectrum showed that DICI+chemotherapy ranked first for overall population and subgroups including squamous, non-squamous, any level of PD-L1 expression, smoking, male, Eastern Cooperative Oncology Group performance status (ECOG PS) = 0/1, age < 65/≥65 while SICI+CT for low tumor mutation burden (TMB), non-smoking, and female subgroups, and DICI for high TMB subgroups.

**Conclusions:**

In the first-line therapy for advanced wild-type NSCLC, both SICI- and DICI-based treatments could bring significant overall advantages over chemotherapy, with comparable outcomes of efficacy and ≥3AEs. DICI-based treatments were more effective than SICI-based treatments in squamous and PD-L1 <1% subgroups. For most populations, DICI+chemotherapy could be the best choice with a survival benefit, while SICI+chemotherapy has established its position actually.

**Systematic Review Registration:**

[PROSPERO], identifier [CRD42020184534].

## Introduction

Lung cancer is the cancer with the highest mortality worldwide (1), among which non-small cell lung cancer (NSCLC) accounts for approximately 85% (2). Due to that advanced driver-gene wild-type NSCLC cannot benefit from targeted therapy (3), the third-generation platinum-containing chemotherapy (CT) was the standard first-line therapy in the past. Although pemetrexed or bevacizumab (BEV) maintenance therapy has brought survival benefits for non-squamous NSCLC, the 5-year overall survival (OS) rate of late-stage NSCLC is still limited (4). In recent years, with the development of immune checkpoint inhibitors (ICIs), including programmed death 1 (PD-1), programmed death-ligand 1 (PD-L1), and cytotoxic T lymphocyte-associated antigen 4 (CTLA-4) inhibitors (5), the first-line treatments for driver-gene negative advanced NSCLC have been enriched and optimized, significantly extending the survival of patients (6). As for single ICI (SICI), KEYNOTE 024 proved that pembrolizumab (PEM) significantly increased OS and progression-free survival (PFS) in advanced wild-type NSCLC patients with PD-L1 ≥50% (7, 8). In addition, IMpower 110 demonstrated atezolizumab (ATE) significantly prolonged OS in patients with Tumor cell/Immune cell (TC/IC) = 3 (9). Recently, EMPOWER-LUNG1 also demonstrated that cemiplimab (CEM) prolonged patients’ OS and PFS in PD-L1 ≥50% significantly (10). For patients with a low expression of PD-L1, SICI plus CT (SICI+CT) showed better efficacy. KEYNOTE 021 (11, 12), KEYNOTE 189 (13, 14), KEYNOTE 407 (15, 16), CheckMate 227 part2 (17), CAMEL (18), ORIENT-11 (19), and ORIENT-12 (20) evaluated the efficacy of PD-1 inhibitors in combination with platinum-based CT and obtained significant benefits. However, great differences exist in the efficacy of anti-PD-L1 or CTLA-4 antibodies combined with CT in randomized controlled trials (RCTs) such as IMpower 130 (21), IMpower 131 (22), IMpower 132 (23, 24), Govindan (25), and Lynch (26). In addition to SICI-based treatments (including SICI and SICI+CT), dual ICIs (DICI)-based treatments have also been meaningfully explored. CheckMate 227 proved that nivolumab (NIV) combined with ipilimumab (IPI) improved OS and PFS in patients with advanced wild-type NSCLC (27). Furthermore, durvalumab (DUR) combined with tremelimumab (TRE) failed to indicate OS advantage over CT and is even inferior to CT in PFS in MYSTIC (28). CheckMate 9LA was the first study proving that DICI combined with CT (DICI+CT) significantly improved efficacy; NIV+IPI+CT gained longer OS and PFS over CT (29). While in CCTG BR.34, DUR+TRE+CT failed to obtain OS advantage in contrast to DICI (30).

Both SICI-based and DICI-based treatments have achieved certain success. However, no studies have been conducted to compare the two treatments directly. In theory, DICI-based treatments could target more immune checkpoints and should be more effective but may also produce more side effects. It has become a huge challenge perplexing clinicians whether DICI-based therapies are more effective and whether there exists the best treatment or beneficial populations among SICI, SICI+CT, DICI, and DICI+CT. To address such questions reasonably, we conducted an integrated analysis and network meta-analysis (NMA). Our study summarized the general effects of related treatments and compared the efficacy and safety among SICI, SICI+CT, DICI, DICI+CT, and CT in the first-line treatment of advanced wild-type NSCLC, which will provide valuable evidence for clinical decision-making.

## Materials and Methods

### Literature Searching Strategies

This NMA was performed according to the PRISMA extension statement (**Supplementary Table 1**). We used strategies in **Supplementary Table 2** to search literature on first-line immunotherapy of advanced wild-type NSCLC in PubMed, Embase, and the Cochrane Central Register of Controlled Trials (January 1, 2005–December 31, 2020). Abstracts of major international oncology conferences (American Society of Clinical Oncology, European Society of Medical Oncology, and World Conference on Lung Cancer) were also reviewed (2018–2020).

### Inclusion Criteria

Published phase II/III RCTs reported in English that compared at least two first-line treatments, at least one arm containing ICIs, for histologically confirmed advanced (stage III–IV) driver-gene wild-type NSCLC patients who did not receive prior systemic therapies. The hazard ratio (HR) and 95% confidence interval (CI) of OS and PFS are available.

### Exclusion Criteria

Trials involving targeted therapy for driver-gene mutation NSCLC patients or therapies other than ICIs or CT, such as surgery, radiotherapy, antiangiogenesis, immune cells, and cancer vaccines, or currently unavailable drugs such as the anti-TIGIT antibody tiragolumab. Trials that only reported outcomes of maintenance therapy were also excluded.

### Data Extraction and Risk of Bias Assessment

We extracted study name, first author, publication year, number and characteristics of patients, OS, PFS, objective response rate (ORR), and the incidence of grade 3 or higher adverse events (≥3AEs) related to treatments. For the same study that reported outcomes of different follow-up times, we extracted the most recent data.

We assessed the bias risk of RCTs using the Cochrane Risk of Bias Tool, including seven items: random sequence generation, allocation concealment, blinding of participants and personnel, blinding of outcome assessment, incomplete outcome data, selective outcome reporting, and other sources of bias (31). RCTs can be evaluated as low, high, or ambiguous risk of bias. Data extraction and risk of bias assessment were conducted by two independent investigators (QX and XZ).

### Data Analysis

To judge the median OS (mOS) of each treatment tentatively, we performed pairwise meta-analyses with the frequentist method for head-to-head trials. Heterogeneity between studies was assessed using the *Q* test and *I* ^2^ statistics. The random model was used when *I* ^2^ ≥ 50 or p < 0.05, in which heterogeneity was considered statistically significant (32).

For survival variables (OS/PFS) and binary variables (ORR/≥3AEs), HR or odds ratio (OR) and corresponding 95% CIs were pooled according to the fixed or random model, which were compared using deviance information criteria (DIC) (33). We used the JAGS and GEMTC package in R.3.6.1 for Bayesian NMA using a Markov Chain Monte Carlo simulation technique. For each outcome, 150,000 sample iterations were generated with 100,000 burn-ins and a thinning interval of 1. To ensure the convergence of the model, visual inspection methods of trace plots and Brooks–Gelman–Rubin diagnostic were adopted (34). We used Stata 16.0 to generate network plots, indicating more directly the relationships between treatments. For network consistency, node splitting analysis was used to evaluate the differences between direct and indirect comparisons in the closed loop of treatments. Transitivity was evaluated using visual graphics for patient characteristics between treatment groups and control groups, respectively. To estimate the probability of each treatment being at each rank, we calculated the surface under the cumulative ranking curve (SUCRA). The higher SUCRA value represents that a treatment is to be ranked on the top more likely (35).

## Results

### Study Characteristics of Network Meta-Analysis

**Figure 1:**
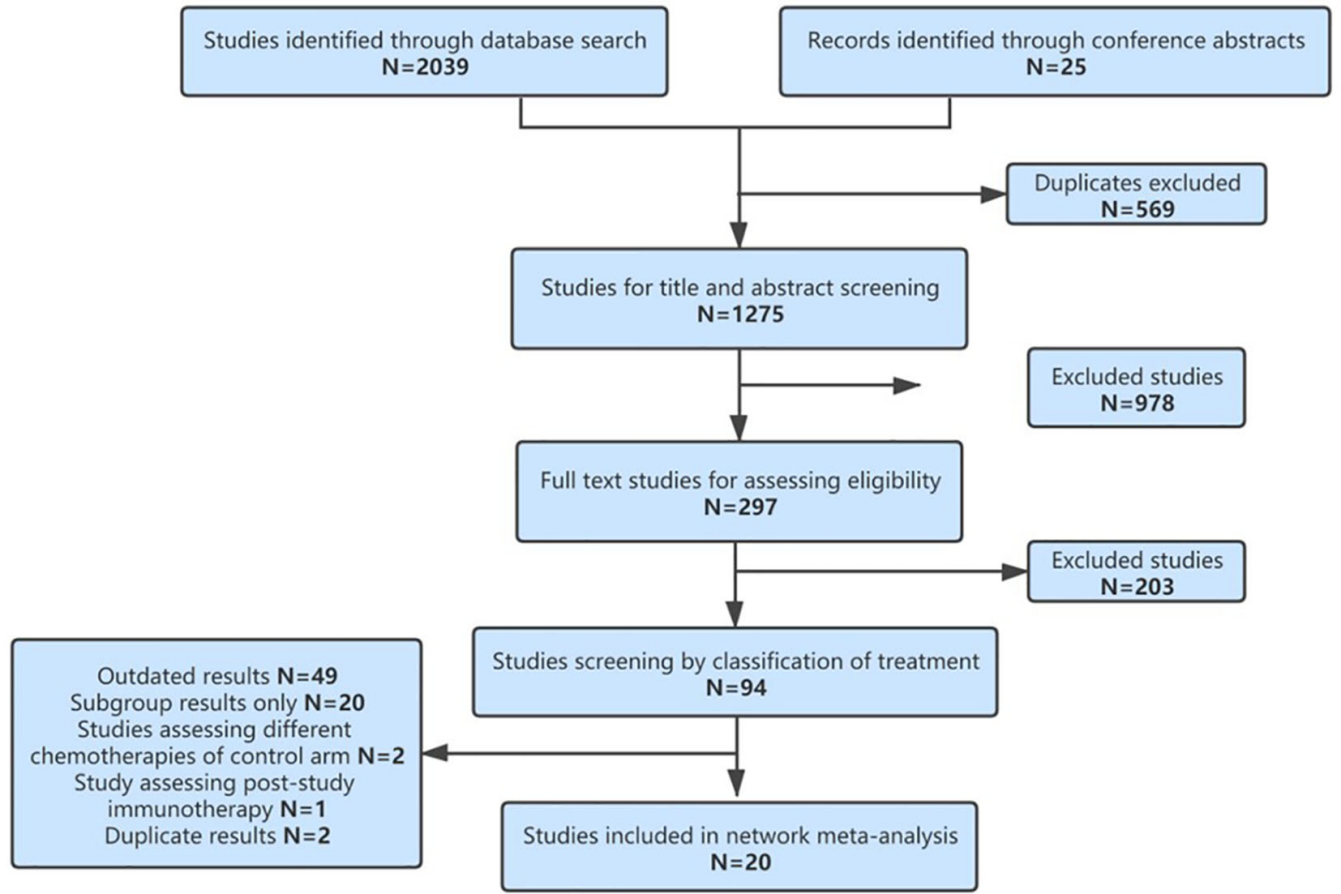
Flowchart of study selection.

According to the study screening process in **Figure 1**, 20 RCTs were eligible for our NMA, including 13,032 patients and 17 different treatments. They are SICI regimens, including PEM (7, 8, 36), CEM (10), NIV (37), ATE (9), and DUR (28); SICI+CT regimens, including PEM+CT (11–16), sintilimab (SIN)+CT (19, 20), ATE+CT (21–24), IPI+CT (25, 26), camrelizumab (CAM)+CT (18), and NIV+CT (17); DICI regimens, including DUR+TRE (28) and NIV+IPI (27, 38); DICI+CT regimens, including DUR+TRE+CT (28, 30) and NIV+IPI+CT (29); and CT as control group, including CT with maintenance with pemetrexed (Mpem) and platinum-based doublet CT. The baseline characteristics of the studies were shown in **Table 1**.

**Table 1:** Baseline characteristics of studies included in the network meta-analysis of patients with wild-type advanced non-small cell lung cancer.

Study name (year)	Phase	Population	Sample size	Median age	Male/Female	Intervention arm	Control arm
**SICI**							
KEYNOTE 024 (2016/2019)	III	squ/non-squ PD-L1≥50%	154/151	65/66	187/118	PEM	CT
KEYNOTE 042 (2019)	III	squ/non-squ PD-L1≥1%	637/637	63/63	902/372	PEM	CT
IMpower 110 (2019)	III	squ/non-squ PD-L1≥1%	277/277	NG/NG	389/165	ATE	CT
MYSTIC (2020)	III	squ/non-squ	374/372	65/64	506/240	DUR	CT
CheckMate 026 (2017)	III	squ/non-squ PD-L1≥1%	271/270	63/65	332/209	NIV	CT
CheckMate 227 part1 (2019)	III	squ/non-squ PD-L1≥1%	396/397	64/64	532/261	NIV	CT
EMPOWER-LUNG1 (2020)	III	squ/non-squ	356/354	63/64	606/104	CEM	CT
**SICI+CT**							
KEYNOTE 021G (2016/2019)	II	non-squ	60/63	63/63	48/75	PEM+CT	CT+Mpem
KEYNOTE 189 (2018/2020)	III	non-squ	410/206	65/64	363/253	PEM+CT	CT+Mpem
KEYNOTE 407 (2018/2020)	III	squ	278/281	65/65	455/104	PEM+CT	CT
IMpower 130 (2019)	III	non-squ	483/240	64/65	415/308	ATE+CT	CT
IMpower 132 (2018/2020)	III	non-squ	292/286	64/63	384/194	ATE+CT	CT+Mpem
IMpower 131 (2020)	III	squ	343/340	65/65	557/126	ATE+CT	CT
CAMEL (2019)	III	non-squ	205/207	59/61	295/117	CAM+CT	CT
ORIENT-12 (2020)	III	squ	179/178	64/62	327/50	SIN+CT	CT
ORIENT-11 (2020)	III	non-squ	266/131	61/61	303/94	SIN+CT	CT
CheckMate 227 part1 (2019)	III	squ/non-squ PD-L1<1%	177/186	64/64	255/108	NIV+CT	CT
CheckMate 227 part2 (2019)	III	squ/non-squ	377/378	63/64	528/227	NIV+CT	CT
Lynch (2012)	II	squ/non-squ	68/66	61/62	98/36	IPI+CT	CT
Govindan (2017)	III	squ	388/361	64/64	635/114	IPI+CT	CT
**DICI**							
MYSTIC (2020)	III	squ/non-squ	372/372	66/64	516/228	DUR+TRE	CT
CheckMate 227 part1 (2019)	III	squ/non-squ PD-L1≥1%	396/397	64/64	515/278	NIV+IPI	CT
CheckMate 227 part1 (2019)	III	squ/non-squ PD-L1<1%	187/186	63/64	263/110	NIV+IPI	CT
**DICI+CT**							
CheckMate 9LA (2020)	III	squ/non-squ	361/358	65/65	503/216	NIV+IPI+CT	CT
**Others**							
CCTG BR.34 (2020)	III	squ/non-squ	151/150	65/63	162/139	DUR+TRE+CT	DUR+TRE

Data are expressed as intervention/control unless indicated otherwise.

Squ, squamous; Non-squ, non-squamous; NG, not given; PEM, pembrolizumab; CEM, cemiplimab; SIN, sintilimab; ATE, atezolizumab; NIV, nivolumab; DUR, durvalumab; TRE, tremelimumab; CAM, camrelizumab; IPI, ipilimumab; CT, chemotherapy; CT+Mpem, CT followed by maintenance with pemetrexed.

The assumption of transitivity was accepted because no variability of population baselines was identified in the treatment group and control group among studies except for KEYNOTE 021 (11, 12), which showed a significant deviation of male proportion (**Supplementary Figure 1**). The risk of bias assessment was summarized in **Supplementary Figure 2**. Model convergence was established in accordance with trace plots and Brooks–Gelman–Rubin diagnostic (**Supplementary Figure 3**).

### Integrated Analysis of Median Overall Survival

**Figure 2:**
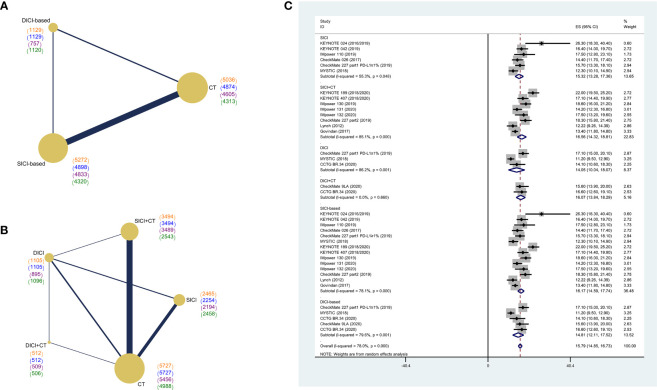
Network diagram of comparisons on different outcomes of treatments and pooled estimates of median overall survival. **(A)** Network diagram of SICI or DICI-based treatments and CT. **(B)** Network diagram of SICI, DICI, SICI+CT, DICI+CT, and CT. Each circular node represents a type of treatment. The size of the nodes and the thickness of the lines are weighted according to the number of studies evaluating each treatment and direct comparison, respectively. The total number of patients receiving treatments was shown in brackets. Color “orange” for overall survival (OS), “blue” for progression-free survival (PFS), “purple” for objective response rate (ORR), “green” for adverse events of grade 3 or higher (≥3AEs). **(C)** Pooled median overall survival (POS) of treatments in the overall population. SICI-based, treatments including single immune checkpoint inhibitor; DICI-based, treatments including double immune checkpoint inhibitors; SICI, single immune checkpoint inhibitor; DICI, double immune checkpoint inhibitors; SICI+CT, single immune checkpoint inhibitor combined with chemotherapy; DICI+CT, double immune checkpoint inhibitors combined with chemotherapy; CT, chemotherapy.

We firstly performed an integrated analysis of mOS in eligible studies to get a pooled OS of current treatment strategies for advanced wild-type NSCLC. The pooled mOS (POS) of ICI-based treatments was 15.79 months (95% CI: 14.85–16.73). The POS of SICI-based treatments was 16.17 months (95% CI: 14.59–17.74), with 15.32 months (95% CI: 13.28–17.36) for SICI and 16.56 months (95% CI: 14.32–18.81) for SICI+CT. The POS of DICI-based treatments was 14.81 months (95% CI: 12.11–17.52), with 14.05 months (95% CI: 10.04–18.07) for DICI and 16.07 months (95% CI: 13.84–18.29) for DICI+CT (**Figure 2C**).

### Network Meta-Analysis of Overall Survival, Progression-Free Survival, Objective Response Rate, Grade 3 or Higher Adverse Events in the Overall Population

**Figure 3:**
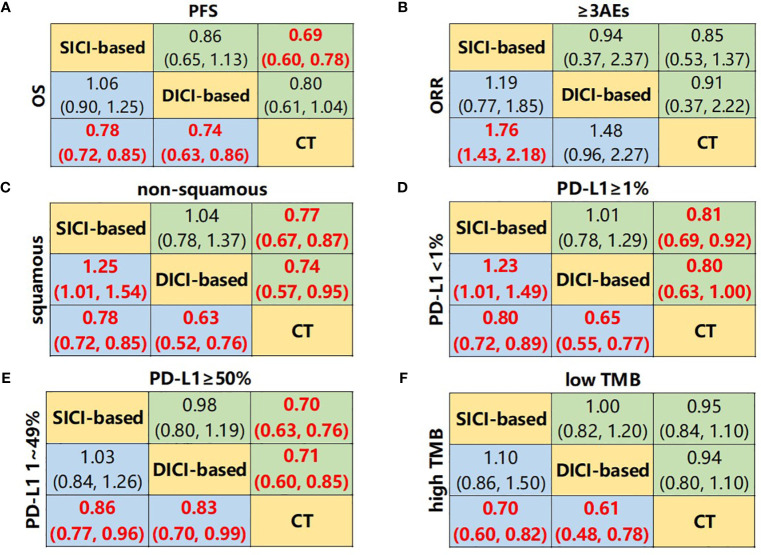
Network meta-analysis composed of SICI- or DICI-based treatments and CT. **(A)** Pooled hazard ratio (HR) [95% CrIs (credible intervals)] for overall survival (OS) and progression-free survival (PFS) in the overall population. **(B)** Pooled odds ratio (OR) (95% CrIs) for objective response rate (ORR) and adverse events of grade 3 or higher (≥3AEs) in the overall population. **(C)** Pooled HR (95% CrIs) for OS of squamous and non-squamous subgroups. **(D)** Pooled HR (95% CrIs) for OS of PD-L1 <1% and PD-L1 ≥1% subgroups. **(E)** Pooled HR (95% CrIs) for OS of PD-L1 1%–49% and PD-L1 ≥50% subgroups. **(F)** Pooled HR (95% CrIs) for OS of high TMB and low TMB subgroups. Data in each cell are HR or OR (95% CrIs) for the comparison of upper row-defining treatment *vs*. lower row-defining treatment. HR less than 1 and OR more than 1 favor upper-row treatment. Significant results are highlighted in red and bold. SICI-based, treatments including single immune checkpoint inhibitor; DICI-based, treatment including double immune checkpoint inhibitors; CT, chemotherapy.

We first compared the difference in efficacy between SICI/DICI-based treatments and CT (**Figure 2A**). Both SICI-based (HR = 0.78, 95% CI: 0.72–0.85) and DICI-based (HR = 0.74, 95% CI: 0.63–0.86) treatments showed significant benefits over CT in mOS, while only SICI-based treatments were superior to CT on median PFS (mPFS) (HR = 0.69, 95% CI: 0.60–0.78) and ORR (OR = 1.76, 95% CI: 1.43–2.18). There were no statistical differences in mOS, mPFS, ORR, and ≥3AEs between SICI-based and DICI-based treatments (**Figures 3A, B**).

**Figure 4:**
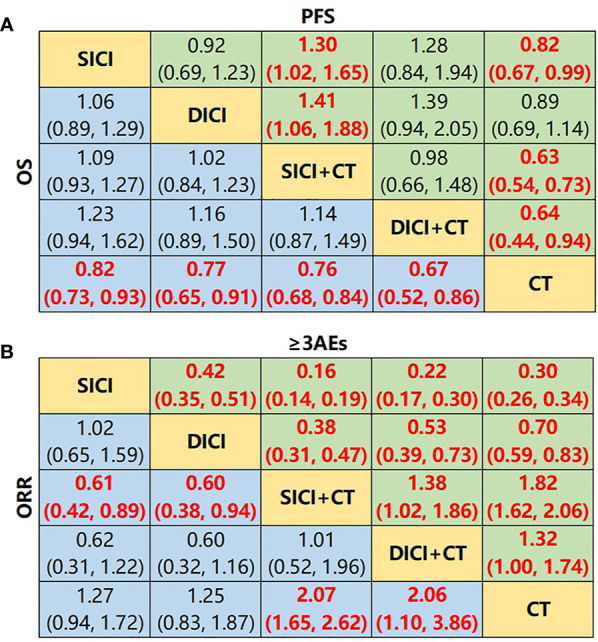
Network meta-analysis of SICI, DICI, SICI+CT, DICI+CT, and CT. **(A)** Pooled hazard ratio (HR) [95% CrIs (credible intervals)] for overall survival (OS) and progression-free survival (PFS) in the overall population. **(B)** Pooled odds ratio (OR) (95% CrIs) for objective response rate (ORR) and adverse events of grade 3 or higher (≥3AEs) in the overall population. Data in each cell are HR or OR (95% CrIs) for the comparison of upper row-defining treatment *vs*. lower row-defining treatment. HR less than 1 and OR more than 1 favor upper-row treatment. Significant results are highlighted in red and bold. SICI, single immune checkpoint inhibitor; DICI, double immune checkpoint inhibitors; SICI+CT, single immune checkpoint inhibitor combined with chemotherapy; DICI+CT, double immune checkpoint inhibitors combined with chemotherapy; CT, chemotherapy.

We then compared the difference in efficacy among SICI, SICI+CT, DICI, DICI+CT, and CT (**Figure 2B**). For mOS, SICI (HR = 0.82, 95% CI: 0.73–0.93), DICI (HR = 0.77, 95% CI: 0.65–0.91), SICI+CT (HR = 0.76, 95% CI: 0.68–0.84), and DICI+CT (HR = 0.67, 95% CI: 0.52–0.86) showed better efficacy over that of CT, but there was no significant difference among the four treatments. For mPFS, SICI (HR = 0.82, 95% CI: 0.67–0.99), SICI+CT (HR = 0.63, 95% CI: 0.54–0.73), and DICI+CT (HR = 0.64, 95% CI: 0.44–0.94) showed significant advantages compared with CT; the efficacy of SICI (HR = 1.30, 95% CI: 1.02–1.65) and DICI (HR = 1.41, 95% CI: 1.06–1.88) was significantly lower than that of SICI+CT (**Figure 4A**).

For ORR, SICI+CT (OR = 2.07, 95% CI: 1.65–2.62) and DICI+CT (OR = 2.06, 95% CI: 1.10–3.86) showed superior efficacy over that of CT. In general, the ORRs of SICI (OR = 0.61, 95% CI: 0.42–0.89) and DICI (OR = 0.60, 95% CI: 0.38–0.94) were lower than that of SICI+CT (**Figure 4B**). In terms of ≥3AEs, those in SICI+CT and DICI+CT were markedly higher than those in SICI, DICI, and CT, while those in SICI and DICI were significantly lower than that in CT. In addition, the incidence of ≥3AEs was significantly lower in SICI compared with that in DICI (OR = 0.42, 95% CI: 0.35–0.51), while ≥3AEs in SICI+CT were significantly higher than that in DICI+CT (OR = 1.38, 95% CI: 1.02–1.86) (**Figure 4B**).

### Network Meta-Analysis of Pathology Subgroup

**Figure 5:**
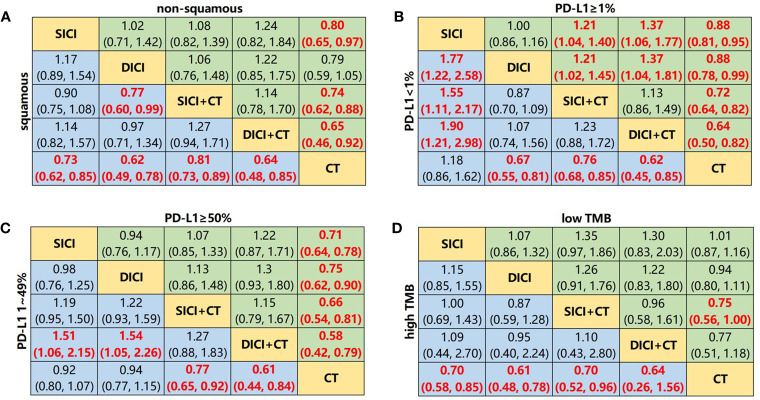
Network meta-analysis for overall survival of subgroup analyses. **(A)** Pooled hazard ratio (HR) [95% CrIs (credible intervals)] for overall survival (OS) of squamous and non-squamous subgroups. **(B)** Pooled HR (95% CrIs) for OS of PD-L1 <1% and PD-L1 ≥1% subgroups. **(C)** Pooled HR (95% CrIs) for OS of PD-L1 1%–49% and PD-L1 ≥50% subgroups. **(D)** Pooled HR (95% CrIs) for OS of high TMB and low TMB subgroups. Data in each cell are HR (95% CrIs) for the comparison of upper row-defining treatment *vs*. lower row-defining treatment. HR less than 1 favors upper row-defining treatment. Significant results are highlighted in red and bold. SICI, single immune checkpoint inhibitor; DICI, double immune checkpoint inhibitors; SICI+CT, single immune checkpoint inhibitor combined with chemotherapy; DICI+CT, double immune checkpoint inhibitors combined with chemotherapy; CT, chemotherapy.

In the squamous NSCLC subgroup, both SICI-based treatments and DICI-based treatments achieved significant OS advantages compared to CT only, while SICI-based treatments achieved significantly shorter mOS than that in DICI-based treatments (HR = 1.25, 95% CI: 1.01–1.54) (**Figure 3C** and **Supplementary Figure 4A**). SICI (HR = 0.73, 95% CI: 0.62–0.85), DICI (HR = 0.62, 95% CI: 0.49–0.78), SICI+CT (HR = 0.81, 95% CI: 0.73–0.89), and DICI+CT (HR = 0.64, 95% CI: 0.48–0.85) showed improved OS over that of CT. In the comparison of these four measures, the mOS of DICI was significantly longer than that of SICI+CT (HR = 0.77, 95% CI: 0.60–0.99) (**Figure 5A**). In terms of mPFS, SICI (HR = 0.57, 95% CI: 0.36–0.89) and SICI+CT (HR = 0.63, 95% CI: 0.45–0.82) showed significant benefits compared with that of CT (**Supplementary Figure 5A**). In non-squamous NSCLC, both SICI-based treatments and DICI-based treatments prolonged OS significantly compared with CT, with no difference between SICI-based and DICI-based treatments. SICI (HR = 0.80, 95% CI: 0.65–0.97), SICI+CT (HR = 0.74, 95% CI: 0.62–0.88), and DICI+CT (HR = 0.65, 95% CI: 0.46–0.92) showed significant OS advantages compared with CT, but DICI failed to prolong OS significantly *vs*. CT (HR = 0.79, 95% CI: 0.59–1.05) (**Figure 5A**); significant PFS benefits were achieved in SICI+CT (HR = 0.59, 95% CI: 0.47–0.74) and DICI+CT (HR = 0.34, 95% CI: 0.13–0.84) (**Supplementary Figure 5A**).

### Network Meta-Analysis of Programmed Death-Ligand 1 Expression Subgroup

In all PD-L1 expression subgroups, SICI-based and DICI-based treatments could prolong OS over CT (**Figures 3D, E** **)**. In PD-L1 <1% subgroup, the OS of SICI-based treatments turned out to be significantly shorter than that in DICI-based treatments (HR = 1.23, 95% CI: 1.01–1.49) (**Figure 3D**). DICI (HR = 0.67, 95% CI: 0.55–0.81), SICI+CT (HR = 0.76, 95% CI: 0.68–0.85), and DICI+CT (HR = 0.62, 95% CI: 0.45–0.85) were obviously better than CT in mOS, while the efficacy of SICI was significantly worse than those of DICI (HR = 1.77, 95% CI: 1.22–2.58), SICI+CT (HR = 1.55, 95% CI: 1.11–2.17), and DICI+CT (HR = 1.90, 95% CI: 1.21–2.98) (**Figure 5B**). In terms of mPFS, DICI, SICI+CT, and DICI+CT also showed significant advantages over CT (**Supplementary Figure 5B**). In PD-L1 ≥1% subgroup, SICI (HR = 0.88, 95% CI: 0.81–0.95), DICI (HR = 0.88, 95% CI: 0.78–0.99), SICI+CT (HR = 0.72, 95% CI: 0.64–0.82), and DICI+CT (HR = 0.64, 95% CI: 0.50–0.82) all achieved obvious OS benefits compared with CT. In addition, both SICI+CT and DICI+CT were significantly better than SICI or DICI (**Figure 5B**). For mPFS, the advantages of DICI (HR = 0.82, 95% CI: 0.69–0.97), SICI+CT (HR = 0.53, 95% CI: 0.48–0.59), and DICI+CT (HR = 0.60, 95% CI: 0.40–0.91) over CT were maintained, while SICI could equally prolong OS compared with CT (HR = 1.00, 95% CI: 0.92–1.09). SICI+CT was superior to SICI and DICI, while DICI was significantly better than SICI (**Supplementary Figure 5B**).

In PD-L1 1%–49% subgroup, SICI+CT (HR = 0.77, 95% CI: 0.65–0.92) and DICI+CT (HR = 0.61, 95% CI: 0.44–0.84) had a significant OS advantage compared with CT. SICI (HR = 1.51, 95% CI: 1.06–2.15) and DICI (HR = 1.54, 95% CI: 1.05–2.26) had significantly worse mOS than that of DICI+CT. In addition, the effect of SICI+CT on mPFS was more prominent than those of SICI and CT (**Figure 5C** and **Supplementary Figure 5C**). In PD-L1 ≥50% subgroup, the OS benefits of SICI (HR = 0.71, 95% CI: 0.64–0.78), DICI (HR = 0.75, 95% CI: 0.62–0.90), SICI+CT (HR = 0.66, 95% CI: 0.54–0.81), and DICI+CT (HR = 0.58, 95% CI: 0.42–0.79) were conspicuous compared with that of CT, while all the differences disappeared within those four ICI-based therapies (**Figure 5C**). Besides, the mPFS of these four treatments was also significantly longer than that of CT, and the efficacy of SICI was significantly inferior to that of SICI+CT (HR = 1.74, 95% CI: 1.24–2.43) (**Supplementary Figure 5C**).

### Network Meta-Analysis of Tumor Mutation Burden Subgroup

The superiority of SICI-based and DICI-based treatments over CT in OS and PFS was observed in the high TMB subgroup. However, there was no statistical difference between SICI and DICI. In the low TMB subgroup, there was also no statistical difference in mOS and mPFS between SICI-based or DICI-based treatments and CT (**Figure 3F** and **Supplementary Figure 4D**). In the high TMB populations, SICI, DICI, SICI+CT, and DICI+CT showed significant prolongation of both OS and PFS in contrast to those of CT (**Figure 5D** and **Supplementary Figure 5D**). In the low TMB populations, only SICI+CT showed a significant advantage over CT in mOS (HR = 0.75, 95% CI: 0.56–1.00) and mPFS (HR = 0.60, 95% CI: 0.46–0.77). In addition, the mPFS of SICI and DICI was statistically inferior to that of CT (**Figure 5D** and **Supplementary Figure 5D**).

### Network Meta-Analysis of Smoking, Gender, Age, or Eastern Cooperative Oncology Group Subgroup

In smokers, all ICI-based measures significantly prolonged OS compared with CT, and SICI+CT was inferior to DICI+CT (HR = 1.28, 95% CI: 1.05–1.57) (**Supplementary Figure 6A**). In non-smokers, the four ICI-based strategies achieved equal outcomes on OS with CT (**Supplementary Figure 6B**). In males, they yielded superior OS than CT, while DICI is the same with DICI+CT (HR = 1.01, 95% CI: 0.82–1.26). DICI was significantly better than SICI; DICI and DICI+CT were also superior to SICI+CT (**Supplementary Figure 7A**).

DICI, SICI+CT, and DICI+CT all showed significant OS benefits compared with CT regardless of age (**Supplementary Figures 8A, B**). In patients <65 years old, the mOS of SICI+CT was significantly shorter than that of DICI+CT (HR = 1.29, 95% CI: 1.00–1.67) (**Supplementary Figure 8B**). In Eastern Cooperative Oncology Group performance status (ECOG PS) = 0 populations, DICI, SICI+CT, and DICI+CT obtained significantly longer mOS than CT, while DICI+CT dramatically reduced the risk of death by 52% (HR = 0.48, 95% CI: 0.32–0.72). When combined with CT, the efficacy of SICI+CT was significantly worse than that of DICI+CT (HR = 1.70, 95% CI: 1.10–2.63) (**Supplementary Figure 9A**). In the ECOG PS = 1 subgroup, SICI, DICI, SICI+CT, and DICI+CT all achieved significant OS benefits compared with CT, while there were no statistical differences among the four ICI-based measures (**Supplementary Figure 9B**).

### Rank Probabilities

The Bayesian ranking curves of comparable treatments in different populations are shown in **Supplementary Figures S10A, B** (ranking profiles and corresponding SUCRA are shown in **Supplementary Figures 11A, B** and **Supplementary Figures 12A, B**). The result of Bayesian ranking is approximately consistent with NMA. Overall, DICI+CT was most likely to be ranked first for mOS; SICI+CT was ranked first for mPFS and ORR (**Supplementary Figure 10A**). In subgroup analysis, mOS of DICI+CT ranked first for squamous, non-squamous, any PD-L1 expression, smoking, males, ECOG PS = 0/1, age <65/≥65; SICI+CT for low TMB, non-smoking, and females; DICI for high TMB (**Supplementary Figures 11A, B**; **Supplementary Figures 12A, B**).

### Inconsistency Assessment and Sensitivity Analyses

The fit of the consistency model in most comparisons was better than that of the inconsistency model, except for mOS (overall, non-squamous, females subgroups), mPFS (overall, squamous, non-squamous, PD-L1 ≥50% subgroups), and ORR, for which the random model was used (**Supplementary Table 3**). Inconsistency between direct and indirect comparisons using the node-splitting approach did not show significant differences in comparisons except for mOS and mPFS in the low TMB subgroup (**Supplementary Table 4**).

The populations of KEYNOTE 024, CheckMate 227, MYSTIC, IMpower 110, and EMPOWER-LUNG1 were all highly PD-L1 selected, which magnified the efficacy of SICI or DICI. Therefore, we conducted sensitivity analysis excluding studies with highly selected populations in overall and squamous, non-squamous subgroups. Sensitivity analysis showed that the NMA results were relatively stable except for some small changes such as in mOS, SICI was significantly worse than SICI+CT (HR = 1.15, 95% CI: 1.04–1.28) and DICI+CT (HR = 1.29, 95% CI: 1.08–1.54); DICI was also inferior to DICI+CT (HR = 1.24, 95% CI: 1.04–1.47). In mPFS, both SICI (HR = 1.00, 95% CI: 0.82–1.23) and DICI (HR = 1.00, 95% CI: 0.78–1.28) were equally effective compared with CT, and the two treatments were inferior to ICI+CT (**Supplementary Figure 13A**). In the non-squamous subgroup, the significant OS advantage of SICI over CT disappeared, while SICI was significantly worse than CT on mPFS (**Supplementary Figures 13C, D**). In squamous NSCLC, DICI+CT replaced DICI to rank first on OS (**Supplementary Figure 14**).

### Network Meta-Analysis of Specific Treatment Regimens

We compared the efficacy and safety of specific treatment regimens (**Supplementary Figure 15**). SICI-based regimens SIN+CT (HR = 0.59, 95% CI: 0.43–0.81), PEM+CT (HR = 0.67, 95% CI: 0.56–0.80), and CEM (HR = 0.68, 95% CI: 0.53–0.87) and DICI-based regimen NIV+IPI+CT (HR = 0.66, 95% CI: 0.55–0.80) significantly prolonged mOS compared with CT. For mPFS, SIN+CT, PEM+CT, CEM, and ATE+CT showed obvious advantages over CT ± Mpem. For ORR, PEM+CT and NIV+CT were superior to CT ± Mpem, while the advantages of SIN+CT over CT disappeared when compared with CT+Mpem. In terms of ≥3AEs, CT-free treatments showed markedly lower ≥3AEs than CT. Compared with CT, ≥3AEs in combination treatments were significantly higher except for DUR+TRE+CT (HR = 0.70, 95% CI: 0.33–1.51), PEM+CT (HR = 1.25, 95% CI: 0.92–1.70), and SIN+CT (HR = 1.19, 95% CI: 0.84–1.67) (**Supplementary Figures 16A, B**).

## Discussion

As mentioned above, to compare and evaluate the efficacy of SICI- and DICI-based therapies in advanced wild-type NSCLC, we performed an integrated analysis of survival outcomes and NMA among these first-line treatment strategies. Despite those negative primary endpoints of many ICI-related RCTs, we found that ICI-based therapies could provide a POS of nearly 16 months for overall patients with advanced NSCLC. Furthermore, both SICI-based therapies (POS: 16.17 months) and DICI-based therapies (POS: 14.81 months) had significant OS benefits compared with CT, without significant difference in mOS, mPFS, ORR, and ≥3AEs between the two ICI-based strategies. DICI-based therapies were significantly superior to SICI-based therapies in squamous and PD-L1 <1% subgroups on mOS. DICI was more effective than SICI in PD-L1 <1% and male subgroups. In subgroups such as smoking, male, age <65, ECOG PS = 0, DICI+CT obtained significantly longer OS than SICI+CT. Bayesian ranking spectrum showed that DICI+CT had the best OS advantage in the overall population and squamous, non-squamous, any PD-L1 level, smoking, male, ECOG PS = 0/1, <65/≥65 subgroups; SICI+CT ranked first in subgroups of low TMB, non-smoking, and female subgroups, while DICI ranked first in high TMB subgroups.

In our NMA, the overall efficacy of SICI-based and DICI-based therapies was consistent possibly due to the limited number of RCTs on DICI-based therapies with different conclusions. Notably, DICI-based therapies were significantly superior to SICI-based therapies in low immunogenicity subgroups (squamous or PD-L1 <1%), suggesting that dual-target interventions can improve the immune response by transforming the “cold” tumors to “hot” tumors and thereby lead to better efficacy. Interestingly, in populations with potentially high immune responses (smoking, male, <65, ECOG PS = 0), DICI+CT also brought more OS benefits than SICI+CT. In terms of specific treatment regimens, NIV+IPI, with or without CT, all obtained positive survival results and got Food and Drug Administration (FDA) approval, while DUR+TRE ± CT failed to replicate the success of NIV+IPI ± CT. So how to match the anti-PD-1/L1 and anti-CTLA-4 correctly is the key to get the most considerable benefit of DICI. Interestingly, when comparing anti-CTLA-4 plus anti-PD-1 therapy with anti-PD-1 monotherapy, we found that the OS of NIV+IPI was significantly higher than that of NIV monotherapy or DUR monotherapy, which is consistent with the finding of the previous study (39). However, the OS benefit of NIV+IPI *vs*. that of PEM monotherapy is comparable, manifesting that PEM may amplify the efficacy of SICI.

Obviously, further explorations are needed. The key to applying DICI-based treatments reasonably focuses on how to reduce the side effects of anti-CTLA-4 and maximize the efficacy and synergy of ICIs combined with CT. Although the current exploration of DICI-based regimens is still insufficient, with the increasing number of related studies and the effective control of drug dose and toxicities, such strategy possesses great potential to improve the survival of patients with advanced NSCLC to a large extent. For example, some novel anti-PD-L1 antibodies, such as M7824 (40) and YM101 (41), exhibited broader ranges of antitumor spectrum compared to the SICI recently. These biologicals simultaneously blocked transforming growth factor (TGF)-β and PD-L1 pathways, or targeted some new immune checkpoints other than PD-1/L1 or CTLA-4, thus having potential to overcome resistance to SICIs or the present DICI treatment in future clinical practices.

We found that SICI-based therapies also obtained satisfactory results. Due to a large number of such studies and participants involved, the integrated results and NMA comparison were more reliable and robust. Based on the current comparative results, SICI-based therapies, especially SICI+CT, were the first-line treatment regimen with definite efficacy and tolerable side effects. In terms of specific treatment regimens, SIN+CT and PEM+CT ranked in the top on OS, with equal ≥3AEs to that of CT alone. Therefore, SICI+CT is currently the most practical treatment for the unscreened population. How to optimize the period and duration of medication to achieve the unity of efficacy improvement and side effect reduction remains a key problem to be resolved.

Our study also has several limitations. First, some studies were classified as moderate or high risk of bias because of inadequate randomization, allocation concealment, and blinding. Second, although all the studies in our analysis included patients with advanced wild-type NSCLC, some studies included a few patients with driver-gene mutated NSCLC. Thirdly, mOS data in some studies were immature and were extracted or calculated from interim analysis or the latest meeting abstracts. Fourth, it is not possible to compare all treatment strategies in each subgroup due to the limited availability of outcomes. For example, the comparison of mPFS in the PD-L1 1%–49% subgroup lacked data on DICI-based therapies. Fifth, the prediction of SUCRA for treatment strategy ranking is not absolute; when SUCRA prediction contradicts NMA results, the HR estimation of NMA should be given priority. Finally, due to the limited number of RCTs and participants involved in DICI-based therapies, the reliability and robustness of related NMA results and conclusions need to be further verified.

## Conclusions

In the first-line therapy for advanced wild-type NSCLC, both SICI-based and DICI-based treatments could bring significant overall advantages *vs*. CT, with comparable outcomes for mOS and ≥3AEs. DICI-based treatments were more effective than SICI-based treatments in squamous and PD-L1 <1% subgroups, while DICI in combination with CT could be the best first-line choice for most populations. We need more research to further evaluate the efficacy and safety of DICI-based treatments. At the same time, SICI-based therapies have established their position in the current first-line treatment. In addition, NMA and ranking possibilities of specific regimens could provide strong evidence for clinical selection of individualized treatment regimens to maximize survival benefits for related patients.

## Data Availability Statement

The raw data supporting the conclusions of this article will be made available by the authors, without undue reservation.

## Author Contributions

LL is the corresponding author. QX and XZ are joint first authors. LL contributed to the study concept and design. QX and XZ took part in the initial literature search and assessed the eligibilities of feasible studies. QX and XZ interpreted the findings and wrote the first draft of the manuscript. QX, XZ, MH, XD, JG, LS, SL, KH, and JW prepared the figures and tables. LL revised and edited the manuscript. All authors approved the final version of the manuscript. LL is the guarantor of this study and accepts full responsibility for the work, had access to the data, and controlled the decision to publish. The corresponding authors attest that all listed authors meet authorship criteria and that no other person meeting the criteria has been omitted. All authors contributed to the article and approved the submitted version.

## Funding

This work was supported by the National Natural Science Foundation of China (81172487 to LL and 81500092 to SL), Natural Science Foundation of Shandong Province (ZR2017MH005 to LL), and Foundation of Shandong University Clinical Research Center (2020SDUCRCC011).

## Conflict of Interest

The authors declare that the research was conducted in the absence of any commercial or financial relationships that could be construed as a potential conflict of interest.

## Publisher’s Note

All claims expressed in this article are solely those of the authors and do not necessarily represent those of their affiliated organizations, or those of the publisher, the editors and the reviewers. Any product that may be evaluated in this article, or claim that may be made by its manufacturer, is not guaranteed or endorsed by the publisher.

## References

[1] Bray F Ferlay J Soerjomataram I Siegel RL Torre LA Jemal A. Global Cancer Statistics 2018: GLOBOCAN Estimates of Incidence and Mortality Worldwide for 36 Cancers in 185 Countries. CA Cancer J Clin (2018) 68:394–424. 10.3322/caac.21492 30207593

[2] Herbst RS Morgensztern D Boshoff C. The Biology and Management of non-Small Cell Lung Cancer. Nature (2018) 553:446–54. 10.1038/nature25183 29364287

[3] Lamberti G Andrini E Sisi M Rizzo A Parisi C Di Federico A. Beyond EGFR, ALK and ROS1: Current Evidence and Future Perspectives on Newly Targetable Oncogenic Drivers in Lung Adenocarcinoma. Crit Rev Oncol Hematol (2020) 156:103119. 10.1016/j.critrevonc.2020.103119 33053439

[4] Goldstraw P Chansky K Crowley J Rami-Porta R Asamura H Eberhardt WE. The IASLC Lung Cancer Staging Project: Proposals for Revision of the TNM Stage Groupings in the Forthcoming (Eighth) Edition of the TNM Classification for Lung Cancer. J Thorac Oncol (2016) 11:39–51. 10.1016/j.jtho.2015.09.009 26762738

[5] Iwai Y Ishida M Tanaka Y Okazaki T Honjo T Minato N. Involvement of PD-L1 on Tumor Cells in the Escape From Host Immune System and Tumor Immunotherapy by PD-L1 Blockade. Proc Natl Acad Sci U S A (2002) 99:12293–7. 10.1073/pnas.192461099 PMC129438 12218188

[6] Nasser NJ Gorenberg M Agbarya A. First Line Immunotherapy for Non-Small Cell Lung Cancer. Pharmaceut (Basel) (2020) 13:373. 10.3390/ph13110373 PMC7695295 33171686

[7] Reck M Rodríguez-Abreu D Robinson AG Hui R Csőszi T Fülöp A. Pembrolizumab *Versus* Chemotherapy for PD-L1-Positive Non-Small-Cell Lung Cancer. N Engl J Med (2016) 375:1823–33. 10.1056/NEJMoa1606774 27718847

[8] Reck M Rodríguez-Abreu D Robinson AG Hui R Csőszi T Fülöp A. Updated Analysis of KEYNOTE-024: Pembrolizumab *Versus* Platinum-Based Chemotherapy for Advanced Non-Small-Cell Lung Cancer With PD-L1 Tumor Proportion Score of 50% or Greater. J Clin Oncol (2019) 37:537–46. 10.1200/JCO.18.00149 30620668

[9] Herbst RS Giaccone G de Marinis F Reinmuth N Vergnenegre A Barrios CH. Atezolizumab for First-Line Treatment of PD-L1-Selected Patients with NSCLC. N Engl J Med (2020) 383:1328–39. 10.1056/NEJMoa1917346 32997907

[10] Sezer A Kilickap S Gümüş M Bondarenko I Özgüroğlu M Gogishvili M. LBA52 EMPOWER-Lung 1: Phase III First-Line (1L) Cemiplimab Monotherapy *vs* Platinum-Doublet Chemotherapy (Chemo) in Advanced Non-Small Cell Lung Cancer (NSCLC) With Programmed Cell Death-Ligand 1 (PD-L1) ≥50%. Ann Oncol Annals Oncol (2020) 31:S1182. 10.1016/j.annonc.2020.08.2285

[11] Langer CJ Gadgeel SM Borghaei H Papadimitrakopoulou VA Patnaik A Powell SF. Carboplatin and Pemetrexed With or Without Pembrolizumab for Advanced, non-Squamous non-Small-Cell Lung Cancer: A Randomised, Phase 2 Cohort of the Open-Label KEYNOTE-021 Study. Lancet Oncol (2016) 17:1497–508. 10.1016/S1470-2045(16)30498-3 PMC6886237 27745820

[12] Borghaei H Langer CJ Gadgeel S Papadimitrakopoulou VA Patnaik A Powell SF. 24-Month Overall Survival From KEYNOTE-021 Cohort G: Pemetrexed and Carboplatin With or Without Pembrolizumab as First-Line Therapy For Advanced Nonsquamous Non-Small Cell Lung Cancer. J Thorac Oncol (2019) 14:124–9. 10.1016/j.jtho.2018.08.004 30138764

[13] Gandhi L Rodríguez-Abreu D Gadgeel S Esteban E Felip E De Angelis F. Pembrolizumab Plus Chemotherapy in Metastatic Non-Small-Cell Lung Cancer. N Engl J Med (2018) 378:2078–92. 10.1056/NEJMoa1801005 29658856

[14] Gadgeel S Rodríguez-Abreu D Speranza G Esteban E Felip E Dómine M. Updated Analysis From KEYNOTE-189: Pembrolizumab or Placebo Plus Pemetrexed and Platinum for Previously Untreated Metastatic Nonsquamous Non-Small-Cell Lung Cancer. J Clin Oncol (2020) 38:1505–17. 10.1200/JCO.19.03136 32150489

[15] Paz-Ares L Luft A Vicente D Tafreshi A Gümüş M Mazières J. Pembrolizumab Plus Chemotherapy for Squamous Non-Small-Cell Lung Cancer. N Engl J Med (2018) 379:2040–51. 10.1056/NEJMoa1810865 30280635

[16] Paz-Ares L Vicente D Tafreshi A Robinson A Soto Parra H Mazières J. A Randomized, Placebo-Controlled Trial of Pembrolizumab Plus Chemotherapy in Patients With Metastatic Squamous NSCLC: Protocol-Specified Final Analysis of KEYNOTE-407. J Thorac Oncol (2020) 15:1657–69. 10.1016/j.jtho.2020.06.015 32599071

[17] Paz-Ares TEC L Yu ea X. Nivolumab (NIVO) 1 Platinum-Doublet Chemotherapy (Chemo) *vs* Chemo as First-Line (1L) Treatment (Tx) for Advanced non-Small Cell Lung Cancer (aNSCLC): CheckMate 227 - Part 2 Final Analysis. Ann Oncol (2019) 30:xi67. 10.1093/annonc/mdz453

[18] Zhou GC C Huang ea Y. A Randomized Phase 3 Study of Camrelizumab Plus Chemotherapy as 1st Line Therapy for Advanced/Metastatic Non-Squamous Non-Small Cell Lung Cancer. J Thorac Oncol (2019) 14:305–14. 10.1016/S2213-2600(20)30365-9

[19] Yang Y Wang Z Fang J Yu Q Han B Cang S. Efficacy and Safety of Sintilimab Plus Pemetrexed and Platinum as First-Line Treatment for Locally Advanced or Metastatic Nonsquamous NSCLC: A Randomized, Double-Blind, Phase 3 Study (Oncology Program by InnovENT Anti-PD-1-11). J Thorac Oncol (2020) 15:1636–46. 10.1016/j.jtho.2020.07.014 32781263

[20] Zhou C Wu L Fan Y Wang Z Liu L Chen G. LBA56 ORIENT-12: Sintilimab Plus Gemcitabine and Platinum (GP) as First-Line (1L) Treatment for Locally Advanced or Metastatic Squamous Non-Small-Cell Lung Cancer (sqNSCLC). Ann Oncol (2020) 31:S1186. 10.1016/j.annonc.2020.08.2289

[21] West H McCleod M Hussein M Morabito A Rittmeyer A Conter HJ. Atezolizumab in Combination With Carboplatin Plus Nab-Paclitaxel Chemotherapy Compared With Chemotherapy Alone as First-Line Treatment for Metastatic Non-Squamous Non-Small-Cell Lung Cancer (IMpower130): A Multicentre, Randomised, Open-Label, Phase 3 Trial. Lancet Oncol (2019) 20:924–37. 10.1016/S1470-2045(19)30167-6 31122901

[22] Jotte R Cappuzzo F Vynnychenko I Stroyakovskiy D Rodríguez-Abreu D Hussein M. Atezolizumab in Combination With Carboplatin and Nab-Paclitaxel in Advanced Squamous NSCLC (IMpower131): Results From a Randomized Phase III Trial. J Thorac Oncol (2020) 15:1351–60. 10.1016/j.jtho.2020.03.028 32302702

[23] Papadimitrakopoulou V CM Bordoni R. IMpower132: PFS and Safety Results With 1L Atezolizumab + Carboplatin/Cisplatin + Pemetrexed in Stage IV Non-Squamous NSCLC. J Thorac Oncol (2018) 13:S332. 10.1016/j.jtho.2018.08.262

[24] Nishio M Barlesi F Ball S Bordoni R Cobo M Dubray-Longeras P. 375o Final Efficacy Results From IMpower132: First-Line Atezolizumab + Chemotherapy in Patients With Stage IV Non-Squamous NSCLC. Ann Oncol (2020) 31:S1386. 10.1016/j.annonc.2020.10.369

[25] Govindan R Szczesna A Ahn MJ Schneider CP Gonzalez Mella PF Barlesi F. Phase III Trial of Ipilimumab Combined With Paclitaxel and Carboplatin in Advanced Squamous Non-Small-Cell Lung Cancer. J Clin Oncol (2017) 35:3449–57. 10.1200/JCO.2016.71.7629 28854067

[26] Lynch TJ Bondarenko I Luft A Serwatowski P Barlesi F Chacko R. Ipilimumab in Combination With Paclitaxel and Carboplatin as First-Line Treatment in Stage IIIB/IV non-Small-Cell Lung Cancer: Results From a Randomized, Double-Blind, Multicenter Phase II Study. J Clin Oncol (2012) 30:2046–54. 10.1200/JCO.2011.38.4032 22547592

[27] Hellmann MD Paz-Ares L Bernabe Caro R Zurawski B Kim SW Carcereny Costa E. Nivolumab Plus Ipilimumab in Advanced Non-Small-Cell Lung Cancer. N Engl J Med (2019) 381:2020–31. 10.1056/NEJMoa1910231 31562796

[28] Rizvi NA Cho BC Reinmuth N Lee KH Luft A Ahn MJ. Durvalumab With or Without Tremelimumab *vs* Standard Chemotherapy in First-Line Treatment of Metastatic Non-Small Cell Lung Cancer: The MYSTIC Phase 3 Randomized Clinical Trial. JAMA Oncol (2020) 6:661–74. 10.1001/jamaoncol.2020.0237 PMC7146551 32271377

[29] Reck M Manuel Cobo Dols MS Bogdan Zurawski JM Eduardo Richardet JB Enriqueta Felip OJ Aurella Alexandru HS. Nivolumab + Ipilimumab + 2 Cycles of Platinum Doublet Chemotherapy *vs* 4 Cycles Chemo as First-Line Treatment Forstage IV non Small Cell Lung Cancer :CheckMate 9la. J Clin Oncol (2020) 38:suppl 9501-9501. 10.1200/JCO.2020.38.15_suppl.9501

[30] Natasha B Leighl SAL Glenwood D Goss BGMH Martin R Stockler MST. CCTG BR.34: A Randomized Trial of Durvalumab and Tremelimumab +/- Platinum-Based Chemotherapy in Patients With Metastatic (Stage IV) Squamous or Nonsquamous Non-Small Cell Lung Cancer (NSCLC). J Clin Oncol (2020) 38:9502. 10.1200/JCO.2020.38.15_suppl.9502

[31] Nasser M. Cochrane Handbook for Systematic Reviews of Interventions. Am J Public Health (2020) 110:753–4. 10.2105/AJPH.2020.305609

[32] Higgins JP Thompson SG Deeks JJ Altman DG. Measuring Inconsistency in Meta-Analyses. BMJ (2003) 327:557–60. 10.1136/bmj.327.7414.557 PMC192859 12958120

[33] Dias S Welton NJ Caldwell DM Ades AE. Checking Consistency in Mixed Treatment Comparison Meta-Analysis. Stat Med (2010) 29:932–44. 10.1002/sim.3767 20213715

[34] Brooks S. General Methods for Monitoring Convergence of Iterative Simulations. J Comput Graphical Stat (1998) 7:434–55. 10.1080/10618600.1998.10474787

[35] Salanti G Ades AE Ioannidis JP. Graphical Methods and Numerical Summaries for Presenting Results From Multiple-Treatment Meta-Analysis: An Overview and Tutorial. J Clin Epidemiol (2011) 64:163–71. 10.1016/j.jclinepi.2010.03.016 20688472

[36] Mok T Wu YL Kudaba I Kowalski DM Cho BC Turna HZ. Pembrolizumab *Versus* Chemotherapy for Previously Untreated, PD-L1-Expressing, Locally Advanced or Metastatic Non-Small-Cell Lung Cancer (KEYNOTE-042): A Randomised, Open-Label, Controlled, Phase 3 Trial. Lancet (2019) 393:1819–30. 10.1016/S0140-6736(18)32409-7 30955977

[37] Carbone DP Reck M Paz-Ares L Creelan B Horn L Steins M. First-Line Nivolumab in Stage IV or Recurrent Non-Small-Cell Lung Cancer. N Engl J Med (2017) 376:2415–26. 10.1056/NEJMoa1613493 PMC6487310 28636851

[38] Hellmann MD Ciuleanu TE Pluzanski A Lee JS Otterson GA Audigier-Valette C. Nivolumab Plus Ipilimumab in Lung Cancer With a High Tumor Mutational Burden. N Engl J Med (2018) 378:2093–104. 10.1056/NEJMoa1801946 PMC7193684 29658845

[39] Wu K Yi M Qin S Chu Q Zheng X Wu K. The Efficacy and Safety of Combination of PD-1 and CTLA-4 Inhibitors: A Meta-Analysis. Exp Hematol Oncol (2019) 8:26. 10.1186/s40164-019-0150-0 31673481 PMC6815037

[40] Lan Y Zhang D Xu C Hance KW Marelli B Qi J. Enhanced Preclinical Antitumor Activity of M7824, a Bifunctional Fusion Protein Simultaneously Targeting PD-L1 and TGF-β. Sci Transl Med (2018) 10:eaan5488. 10.1126/scitranslmed.aan5488 29343622

[41] Yi M Zhang J Li A Niu M Yan Y Jiao Y. The Construction, Expression, and Enhanced Anti-Tumor Activity of YM101: A Bispecific Antibody Simultaneously Targeting TGF-β and PD-L1. J Hematol Oncol (2021) 14:27. 10.1186/s13045-021-01045-x 33593403 PMC7885589

