# Supplementary material for: Comparison of Efficacy and Safety of Single and Double Immune Checkpoint Inhibitor-Based First-Line Treatments for Advanced Driver-Gene Wild-Type Non-Small Cell Lung Cancer: A Systematic Review and Network Meta-Analysis
Source: Front Immunol. 2021 Aug 16;12:731546. doi: 10.3389/fimmu.2021.731546 (PMC8415225; doi:10.3389/fimmu.2021.731546)
Supplement: Supplementary file 1 [file DataSheet_1.pdf]

Supplementary Material

1 Supplementary Figures

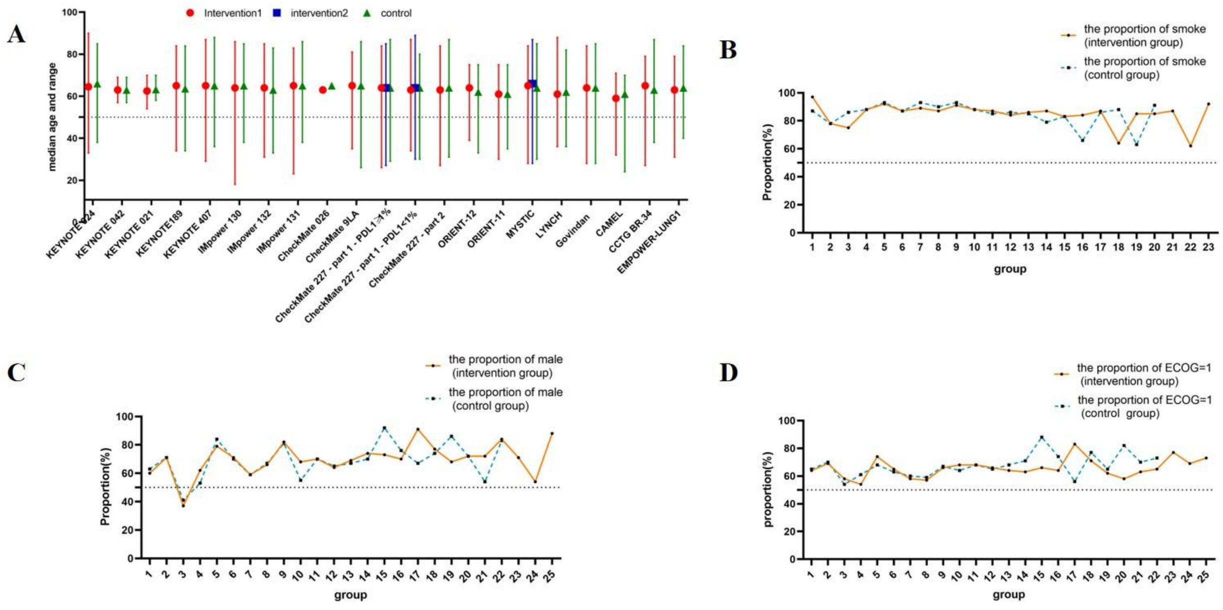

Supplementary Figure 1. Assessment of transitivity. (A) Median age and range of patients in intervention and control groups. (B) The proportion of ever smoking patients in intervention group and control group. (C) The proportion of male patients in intervention group and control group. (D) The proportion of ECOG PS=1 patients in intervention group and control group. Smoke, ever smoking; ECOG PS, Eastern Cooperative Oncology Group Performance Status.

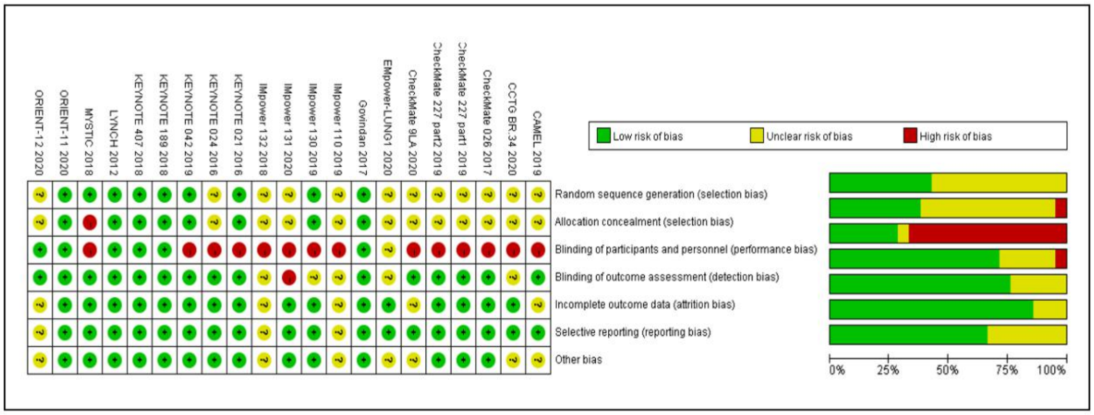

Supplementary Figure 2. Summary of results from bias risk assessment of studies using the Cochrane risk of bias
tool.

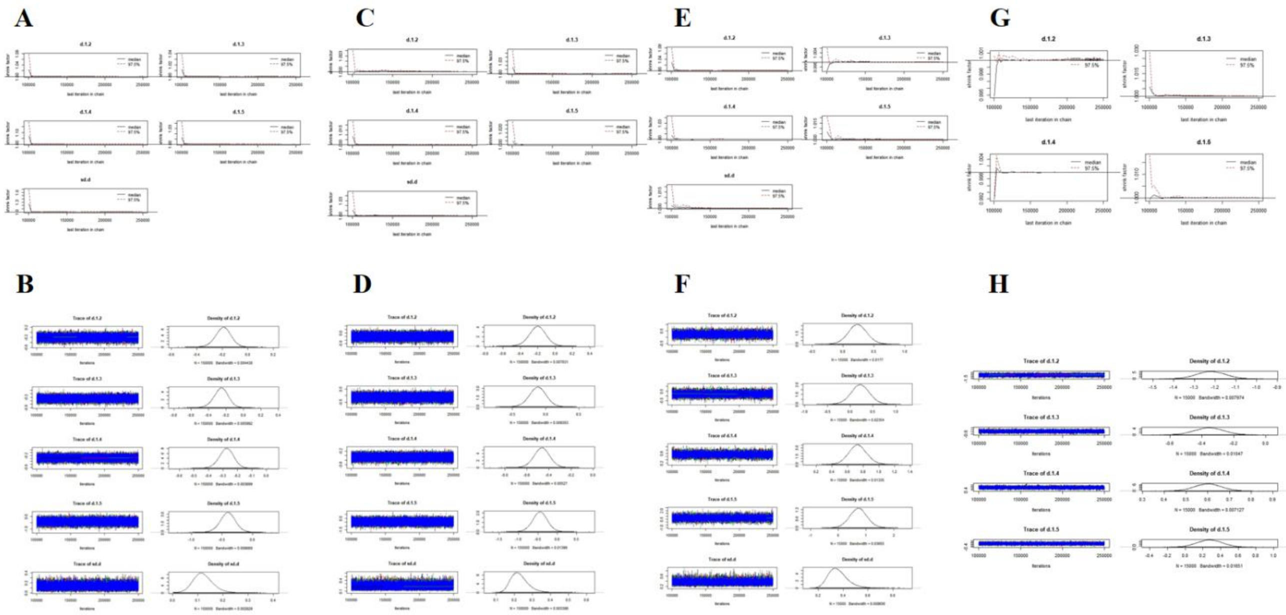

Supplementary Figure 3. Convergence of the four chains established by inspection of the Brooks-Gelman-Rubin diagnostic and the density trace plot. Overall survival (A and B), Progression-free

survival (C and D), Objective response rate (E and F), Adverse events of grade 3 or higher (G and H).

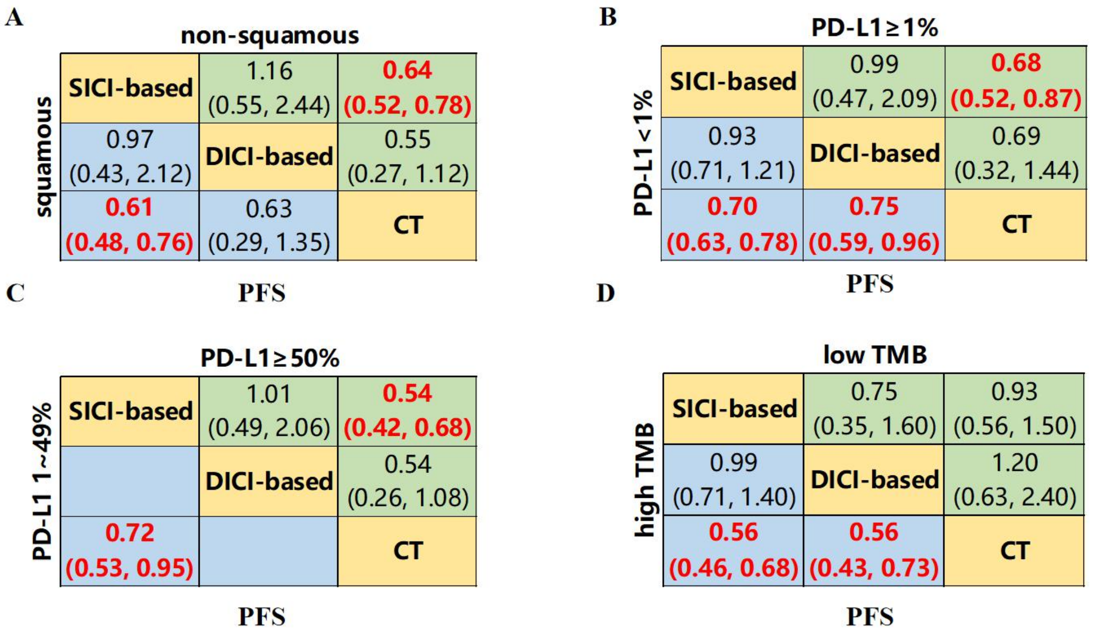

Supplementary Figure 4. Network meta-analysis for progression-free survival of subgroup analyses composed of SICI or DICI-based treatments and CT. (A) Pooled hazard ratio HR (95% CrIs) for progression-free survival (PFS) of squamous and non-squamous subgroups. (B) Pooled HR (95% CrIs) for PFS of PD-L1<1% and PD-L1≥1% subgroups. (C) Pooled HR (95% CrIs) for PFS of PD-L1 1-49% and PD-L1 ≥50% subgroups. (D) Pooled HR (95% CrIs) for PFS of high TMB and low TMB subgroups. Data in each cell are HR (95% CrIs) for the comparison of upper row-defining treatment versus lower row-defining treatment. HR less than 1 favour upper-row treatment. Significant results are highlighted in red and bold. SICI-based, treatments including single immune checkpoint inhibitor; DICI-based, treatment including double immune checkpoint inhibitors; CT, chemotherapy.

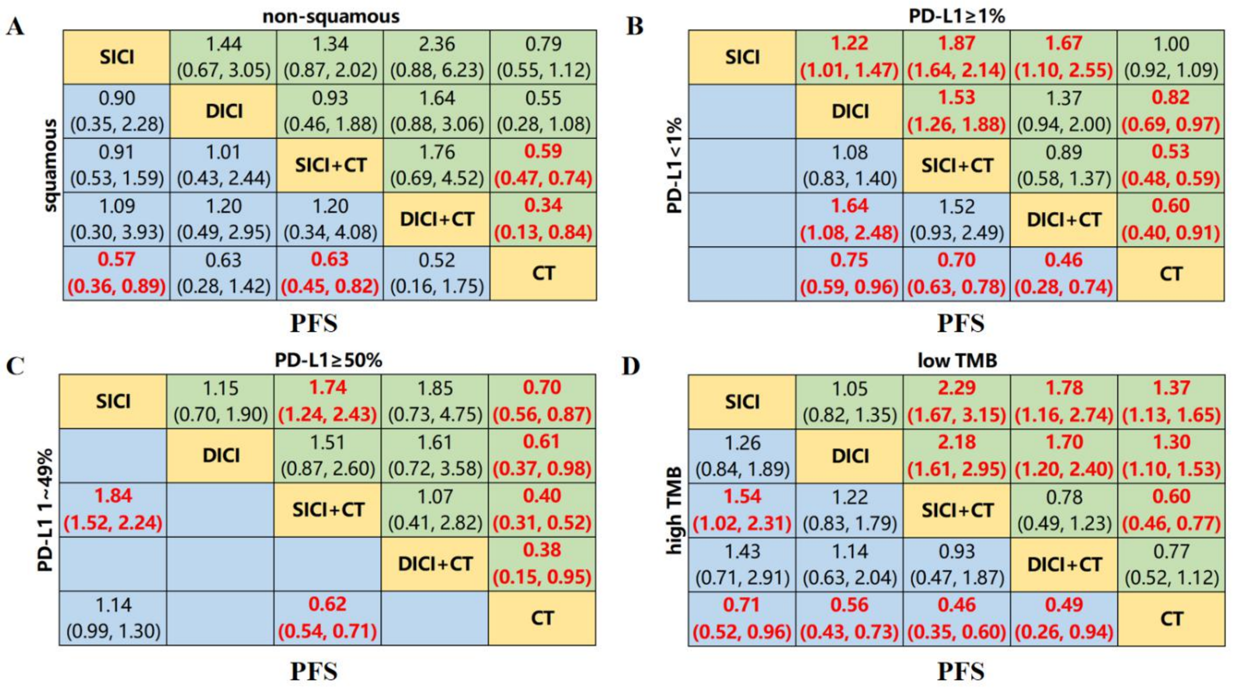

Supplementary Figure 5. Network meta-analysis for progression-free survival of subgroup analyses. (A) Pooled hazard ratio HR (95% CrIs) for progression-free survival (PFS) of squamous and non-squamous subgroups. (B) Pooled HR (95% CrIs) for PFS of PD-L1<1% and PD-L1≥1% subgroups. (C) Pooled HR (95% CrIs) for PFS of PD-L1 1-49% and PD-L1≥50% subgroups. (D) Pooled HR (95% CrIs) for PFS of high TMB and low TMB subgroups. Data in each cell are HR (95% CrIs) for the comparison of upper row-defining treatment versus lower row-defining treatment. HR less than 1 favour upper-row treatment. Significant results are highlighted in red and bold. SICI, Single immune checkpoint inhibitor; DICI, double immune checkpoint inhibitors; SICI+CT, Single immune checkpoint inhibitor combined with chemotherapy; DICI+CT, double immune checkpoint inhibitors combined with chemotherapy; CT, chemotherapy.

A

PFS (upper triangle); OS (lower triangle)

**SICI**		**1.81 (1.42, 2.29)**		1.11 (0.89, 1.38)
1.19 (0.98, 1.43)	**DICI**			
1.08 (0.93, 1.26)	0.91 (0.77, 1.07)	**SICI+CT**		**0.61 (0.56, 0.67)**
**1.39 (1.11, 1.73)**	1.17 (0.96, 1.42)	**1.28 (1.05, 1.57)**	**DICI+CT**	
**0.86 (0.75, 0.97)**	**0.72 (0.63, 0.83)**	**0.79 (0.73, 0.86)**	**0.62 (0.52, 0.74)**	**CT**

Ever smoking

B

PFS (upper triangle); OS (lower triangle)

**SICI**		**4.45 (2.20, 8.96)**		**2.51 (1.30, 4.81)**
0.98 (0.62, 1.55)	**DICI**			
1.22 (0.84, 1.79)	1.25 (0.80, 1.93)	**SICI+CT**		**0.56 (0.44, 0.73)**
1.01 (0.58, 1.76)	1.03 (0.61, 1.74)	0.82 (0.48, 1.42)	**DICI+CT**	
1.00 (0.76, 1.33)	1.02 (0.72, 1.46)	0.82 (0.64, 1.06)	1.00 (0.62, 1.61)	**CT**

Never smoking

Supplementary Figure 6. Network meta-analysis according to smoking history. (A) Pooled hazard ratio (HR) (95% CrIs) for overall survival (OS) and progression-free survival (PFS) in patients with smoking history. (B) Pooled HR (95% CrIs) for OS and PFS in patients without smoking history. Data in each cell are HR (95% CrIs) for the comparison of upper row-defining treatment versus lower row-defining treatment. HR less than 1 favour upper-row treatment. Significant results are highlighted in red and bold. SICI, Single immune checkpoint inhibitor; DICI, double immune checkpoint inhibitors; SICI+CT, Single immune checkpoint inhibitor combined with chemotherapy; DICI+CT, double immune checkpoint inhibitors combined with chemotherapy; CT, chemotherapy.

A

PFS

**SICI**		**1.67 (1.26, 2.21)**		1.05 (0.81, 1.37)
**1.25 (1.01, 1.54)**	**DICI**			
1.02 (0.86, 1.21)	**0.82 (0.68, 0.98)**	**SICI+CT**		**0.63 (0.57, 0.69)**
**1.26 (1.00, 1.61)**	1.01 (0.82, 1.26)	**1.24 (1.00, 1.54)**	**DICI+CT**	
**0.84 (0.73, 0.97)**	**0.68 (0.58, 0.79)**	**0.83 (0.75, 0.91)**	**0.67 (0.55, 0.81)**	**CT**

OS

Male

B

PFS

**SICI**		**2.52 (1.75, 3.61)**		1.36 (0.98, 1.89)
1.09 (0.34, 3.49)	**DICI**			
1.58 (0.65, 3.90)	1.45 (0.54, 3.95)	**SICI+CT**		**0.54 (0.47, 0.62)**
1.55 (0.48, 5.04)	1.42 (0.58, 3.51)	0.98 (0.36, 2.66)	**DICI+CT**	
1.01 (0.47, 2.17)	0.92 (0.38, 2.24)	**0.64 (0.40, 1.00)**	0.65 (0.27, 1.59)	**CT**

OS

Female

Supplementary Figure 7. Network meta-analysis according to gender. (A) Pooled hazard ratio (HR) (95% CrIs) for overall survival (OS) and progression-free survival (PFS) in male patients. (B) Pooled HR (95% CrIs) for OS and PFS in female patients. Data in each cell are HR or (95% CrIs) for the comparison of upper row-defining treatment versus lower row-defining treatment. HR less than 1 favour upper-row treatment. Significant results are highlighted in red and bold. SICI, Single immune checkpoint inhibitor; DICI, double immune checkpoint inhibitors; SICI+CT, Single immune checkpoint inhibitor combined with chemotherapy; DICI+CT, double immune checkpoint inhibitors combined with chemotherapy; CT, chemotherapy.

A

PFS (upper triangle); OS (lower triangle)

**SICI**		**1.93 (1.41, 2.63)**		1.21 (0.91, 1.61)
1.12 (0.87, 1.44)	**DICI**			
1.07 (0.87, 1.32)	0.95 (0.77, 1.18)	**SICI+CT**		**0.63 (0.56, 0.71)**
1.25 (0.94, 1.67)	1.12 (0.87, 1.44)	1.17 (0.90, 1.52)	**DICI+CT**	
0.89 (0.75, 1.06)	**0.79 (0.66, 0.95)**	**0.83 (0.74, 0.93)**	**0.71 (0.56, 0.89)**	**CT**

Age≥65

B

PFS (upper triangle); OS (lower triangle)

**SICI**		**1.98 (1.46, 2.69)**		1.17 (0.88, 1.56)
1.24 (0.97, 1.57)	**DICI**			
1.18 (0.97, 1.44)	0.96 (0.78, 1.18)	**SICI+CT**		**0.59 (0.53, 0.66)**
**1.53 (1.15, 2.03)**	1.24 (0.96, 1.59)	**1.29 (1.00, 1.67)**	**DICI+CT**	
0.89 (0.75, 1.04)	**0.72 (0.60, 0.86)**	**0.75 (0.67, 0.84)**	**0.58 (0.46, 0.73)**	**CT**

Age<65

Supplementary Figure 8. Network meta-analysis according to age. (A) Pooled hazard ratio (HR) (95% CrIs) for overall survival (OS) and progression-free survival (PFS) in age≥65 patients. (B) Pooled HR (95% CrIs) for OS and PFS in age<65 patients. Data in each cell are HR (95% CrIs) for the comparison of upper row-defining treatment versus lower row-defining treatment. HR less than 1 favour upper-row treatment. Significant results are highlighted in red and bold. SICI, Single immune checkpoint inhibitor; DICI, double immune checkpoint inhibitors; SICI+CT, Single immune checkpoint inhibitor combined with chemotherapy; DICI+CT, double immune checkpoint inhibitors combined with chemotherapy; CT, chemotherapy.

A

PFS (upper triangle) / OS (lower triangle)

**SICI**		**3.04 (2.07, 4.45)**		**1.69 (1.18, 2.42)**
1.24 (0.88, 1.75)	**DICI**			
1.07 (0.81, 1.41)	0.86 (0.64, 1.15)	**SICI+CT**		**0.56 (0.49, 0.64)**
**1.81 (1.13, 2.91)**	1.46 (0.90, 2.35)	**1.70 (1.10, 2.63)**	**DICI+CT**	
0.87 (0.69, 1.10)	**0.70 (0.55, 0.90)**	**0.82 (0.70, 0.95)**	**0.48 (0.32, 0.72)**	**CT**

ECOG PS=0

B

PFS (upper triangle) / OS (lower triangle)

**SICI**		**1.61 (1.23, 2.10)**		1.01 (0.79, 1.29)
1.12 (0.91, 1.38)	**DICI**			
1.11 (0.94, 1.31)	0.99 (0.82, 1.20)	**SICI+CT**		**0.63 (0.57, 0.69)**
1.15 (0.88, 1.50)	1.03 (0.78, 1.36)	1.04 (0.81, 1.33)	**DICI+CT**	
**0.86 (0.76, 0.98)**	**0.77 (0.65, 0.91)**	**0.78 (0.70, 0.86)**	**0.75 (0.59, 0.94)**	**CT**

ECOG PS=1

Supplementary Figure 9. Network meta-analysis according to Eastern Cooperative Oncology Group performance status (ECOG PS). (A) Pooled hazard ratio (HR) (95% CrIs) for overall survival (OS) and progression-free survival (PFS) in ECOG PS=0 patients. (B) Pooled HR (95% CrIs) for OS and PFS in ECOG PS=1 patients. Data in each cell are HR (95% CrIs) for the comparison of upper row-defining treatment versus lower row-defining treatment. HR less than 1 favour upper-row treatment. Significant results are highlighted in red and bold. SICI, Single immune checkpoint inhibitor; DICI, double immune checkpoint inhibitors; SICI+CT, Single immune checkpoint inhibitor combined with chemotherapy; DICI+CT, double immune checkpoint inhibitors combined with chemotherapy; CT, chemotherapy.

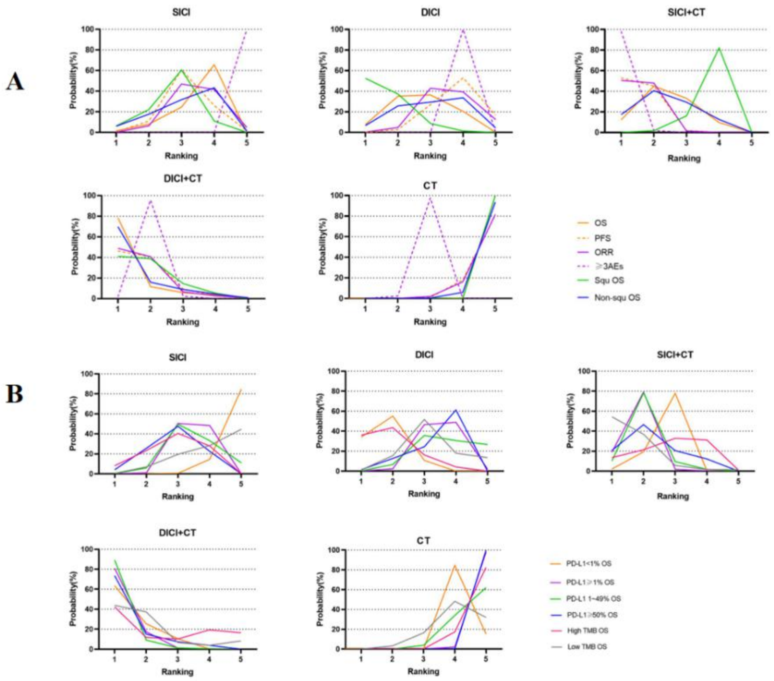

Supplementary Figure 10. Ranking curves indicating the probability of each comparable treatment being ranked from first to last. (A) overall survival (OS), progression-free survival (PFS), objective response rate (ORR), adverse events of grade 3 or higher (≥3 AEs), OS for squamous and non-squamous subgroups. (B) OS for PD-L1 and TMB subgroups. SICI, Single immune checkpoint inhibitor; DICI, double immune checkpoint inhibitors; SICI+CT, Single immune checkpoint inhibitor combined with chemotherapy; DICI+CT, double immune checkpoint inhibitors combined with chemotherapy; CT, chemotherapy; Squ, squamous; Non-squ, non-squamous; PD-L1, programmed-death ligand 1; TMB, tumor mutation burden.

A

	Overall			Histological type		PD-L1				TMB		Smoke		Sex		ECOG PS		Age		Sensitivity analysis				
	OS	ORR	≥3AEs	Squ	Non-squ	<1%	≥1%	1~49%	≥50%	high	low	yes	no	male	female	0	1	≥65	<65	OS	ORR	≥3AEs	Squ	Non-squ
DICI+CT	1	2	2	2	1	1	1	1	1	2	2	1	2	1	2	1	1	1	1	1	2	2	1	1
SICI+CT	2	1	1	4	2	3	2	2	2	3	1	3	1	3	1	3	3	3	3	2	1	1	4	2
DICI	3	4	4	1	3	2	4	4	4	1	3	2	5	2	3	2	2	2	2	3	3	4	2	3
SICI	4	3	5	3	4	5	3	3	3	4	5	4	3	4	4	4	4	4	4	4	4	5	3	4
CT	5	5	3	5	5	4	5	5	5	5	4	5	4	5	5	5	5	5	5	5	5	3	5	5

B

	Overall			Histological type		PD-L1				TMB		Smoke		Sex		ECOG PS		Age		Sensitivity analysis				
	PFS	ORR	≥3AEs	Squ	Non-squ	<1%	≥1%	1~49%	≥50%	high	low	yes	no	male	female	0	1	≥65	<65	PFS	ORR	≥3AEs	Squ	Non-squ
DICI+CT	2	2	2	1	1	1	2	NA	2	2	2	NA	NA	NA	NA	NA	NA	NA	NA	2	2	2	NA	NA
SICI+CT	1	1	1	3	3	2	1	1	1	1	1	1	1	1	1	1	1	1	1	1	1	1	1	1
DICI	4	4	4	4	2	3	3	NA	3	3	4	NA	NA	NA	NA	NA	NA	NA	NA	3	3	4	NA	NA
SICI	3	3	5	2	4	NA	5	3	4	4	5	3	3	3	3	3	3	3	3	5	4	5	2	3
CT	5	5	3	5	5	4	4	2	5	5	3	2	2	2	2	2	2	2	2	4	5	3	3	2

Supplementary Figure 11. Bayesian ranking profiles of comparable treatments on efficacy and safety. (A) Number in each cell indicates the probability of each treatment being ranked from first to last on overall OS, OS for subgroups, ORR and ≥3AEs according to the value of surface under the cumulative ranking curve (SUCRA). (B) Number in each cell indicates the probability of each treatment being ranked from first to last on overall PFS, PFS for subgroups, ORR and ≥3AEs according to the value of SUCRA. SICI, Single immune checkpoint inhibitor; DICI, double immune checkpoint inhibitors; SICI+CT, Single immune checkpoint inhibitor combined with chemotherapy; DICI+CT, double immune checkpoint inhibitors combined with chemotherapy; CT, chemotherapy; Squ, squamous; Non-squ, non-squamous; PD-L1, programmed-death ligand 1; TMB, tumor mutation burden; ECOG PS, Eastern Cooperative Oncology Group Performance Status.

A

		Overall		Histological type			PD-L1			TMB		Smoke		Sex		ECOG PS		Age			Sensitivity analysis			
	OS	ORR	≥3AEs	Squ	Non-squ	<1%	≥1%	1~49%	≥50%	high	low	yes	no	male	female	0	1	≥65	<65	OS	ORR	≥3AEs	Squ	Non-squ
DICI/CT	91	83	75	79	87	88	95	97	90	61	76	98	45	87	74	98	76	90	98	97	84	75	82	94
SICI/CT	65	87	99	30	66	56	79	74	69	54	85	50	85	41	79	46	69	55	58	76	85	100	37	78
DICI	57	35	25	85	49	81	37	31	39	78	43	72	39	85	38	70	70	67	67	45	43	25	79	46
SICI	36	39	0	56	46	4	38	38	53	53	23	30	41	40	31	33	34	36	25	32	24	0	52	24
CT	0	5	51	0	2	21	1	10	0	4	23	0	40	0	27	3	1	2	2	0	14	51	0	8

B

		Overall		Histological type			PD-L1			TMB		Smoke		Sex		ECOG PS		Age			Sensitivity analysis			
	PFS	ORR	≥3AEs	Squ	Non-squ	<1%	≥1%	1~49%	≥50%	high	low	yes	no	male	female	0	1	≥65	<65	PFS	ORR	≥3AEs	Squ	Non-squ
DICI/CT	82	83	75	68	95	98	81	NA	83	73	76	NA	NA	NA	NA	NA	NA	100	100	81	84	75	NA	NA
SICI/CT	88	87	99	56	61	58	93	100	85	85	96	100	100	100	100	100	100	NA	NA	92	85	100	88	100
DICI	29	35	25	53	61	43	50	NA	47	59	16	NA	NA	NA	NA	NA	NA	NA	NA	26	43	25	NA	NA
SICI	46	39	0	67	30	NA	13	2	34	32	9	9	0	18	2	0	24	5	7	25	24	0	46	1
CT	5	5	51	7	3	0	13	48	1	1	52	41	50	32	48	50	26	45	43	25	14	51	15	49

Supplementary Figure 12. Number (percentage of SUCRA) of comparable treatments on efficacy and safety. (A) Number (percentage of surface under the cumulative ranking curve (SUCRA)) in each cell indicates the probability of each treatment being ranked from first (high value) to last (low value) on overall OS, OS for subgroups, ORR, and ≥3AEs. (B) Number (percentage of SUCRA) in each cell indicates the probability of each treatment being ranked from first (high value) to last (low value) on overall PFS, PFS for subgroups, ORR and ≥3AEs. SICI, Single immune checkpoint inhibitor; DICI, double immune checkpoint inhibitors; SICI+CT, Single immune checkpoint inhibitor combined with chemotherapy; DICI+CT, double immune checkpoint inhibitors combined with chemotherapy; CT, chemotherapy; Squ, squamous; Non-squ, non-squamous; PD-L1, programmed-death ligand 1; TMB, tumor mutation burden; ECOG PS, Eastern Cooperative Oncology Group Performance Status.

A

PFS (upper triangle) / OS (lower triangle)

**SICI**	1.00 (0.75, 1.35)	**1.62 (1.28, 2.06)**	**1.48 (1.03, 2.15)**	1.00 (0.82, 1.23)
1.04 (0.91, 1.19)	**DICI**	**1.61 (1.22, 2.14)**	**1.48 (1.06, 2.06)**	1.00 (0.78, 1.28)
**1.15 (1.04, 1.28)**	1.10 (0.97, 1.25)	**SICI+CT**	0.92 (0.65, 1.29)	**0.62 (0.54, 0.70)**
**1.29 (1.08, 1.54)**	**1.24 (1.04, 1.47)**	1.12 (0.94, 1.33)	**DICI+CT**	**0.68 (0.49, 0.93)**
**0.88 (0.82, 0.96)**	**0.85 (0.76, 0.95)**	**0.77 (0.72, 0.82)**	**0.69 (0.59, 0.80)**	**CT**

B

≥3AEs (upper triangle) / ORR (lower triangle)

**SICI**	**0.41 (0.33, 0.50)**	**0.17 (0.14, 0.20)**	**0.22 (0.16, 0.30)**	**0.30 (0.26, 0.34)**
0.82 (0.42, 1.61)	**DICI**	**0.41 (0.33, 0.52)**	**0.55 (0.40, 0.75)**	**0.73 (0.60, 0.89)**
**0.52 (0.32, 0.84)**	0.63 (0.32, 1.26)	**SICI+CT**	1.33 (0.98, 1.81)	**1.77 (1.56, 2.01)**
0.51 (0.24, 1.11)	0.63 (0.30, 1.28)	0.99 (0.47, 2.08)	**DICI+CT**	**1.33 (1.01, 1.75)**
1.08 (0.71, 1.64)	1.32 (0.69, 2.50)	**2.08 (1.62, 2.70)**	**2.11 (1.06, 4.24)**	**CT**

C

non-squamous (upper triangle) / squamous (lower triangle)

**SICI**	1.10 (0.87, 1.39)	**1.28 (1.07, 1.53)**	**1.4 (1.10, 1.79)**	0.95 (0.82, 1.10)
1.15 (0.83, 1.59)	**DICI**	1.16 (0.95, 1.43)	**1.27 (1.03, 1.58)**	0.86 (0.72, 1.03)
0.95 (0.76, 1.17)	0.82 (0.62, 1.09)	**SICI+CT**	1.10 (0.88, 1.36)	**0.74 (0.67, 0.82)**
1.18 (0.83, 1.66)	1.02 (0.73, 1.43)	1.24 (0.92, 1.68)	**DICI+CT**	**0.68 (0.56, 0.82)**
**0.76 (0.63, 0.93)**	**0.67 (0.51, 0.86)**	**0.81 (0.73, 0.89)**	**0.65 (0.49, 0.86)**	**CT**

D

non-squamous (upper triangle) / squamous (lower triangle)

**SICI**	**2.20 (1.71, 2.82)**	**1.29 (1.02, 1.63)**
1.33 (0.57, 3.23)	**SICI+CT**	**0.59 (0.54, 0.64)**
0.83 (0.37, 1.87)	**0.63 (0.45, 0.83)**	**CT**

Supplementary Figure 13. Network meta-analysis excluding highly selected populations. (A) Pooled hazard ratios (HR) (95% CrIs (credible intervals)) for overall survival (OS) and progression-free survival (PFS). (B) Pooled odds ratio (OR) (95% CrIs) for objective response rate (ORR) and adverse events of grade 3 or higher (≥3AEs). (C) Pooled HR (95% CrIs) for OS of squamous and non-squamous subgroups. (D) Pooled HR (95% CrIs) for PFS of squamous and non-squamous subgroups. Data in each cell are HR or OR (95% CrIs) for the comparison of upper row-defining treatment versus lower row-defining treatment. HR less than 1 and OR more than 1 favour upper-row treatment. Significant results are highlighted in red and bold. SICI, Single immune checkpoint inhibitor; DICI, double immune checkpoint inhibitors; SICI+CT, Single immune checkpoint inhibitor combined with chemotherapy; DICI+CT, double immune checkpoint inhibitors combined with chemotherapy; CT, chemotherapy.

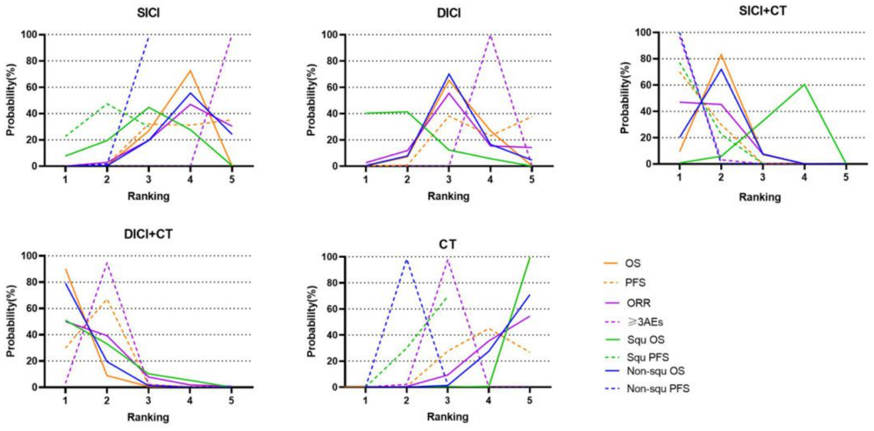

Supplementary Figure 14. Ranking curves indicating the probability of each comparable treatment being ranked from first to last excluding highly selected populations. Overall survival (OS), progression-free survival (PFS), objective response rate (ORR), adverse events of grade 3 or higher (≥3AEs), OS and PFS for squamous and non-squamous subgroups. SICI, Single immune checkpoint inhibitor; DICI, double immune checkpoint inhibitors; SICI+CT, Single immune checkpoint inhibitor combined with chemotherapy; DICI+CT, double immune checkpoint inhibitors combined with chemotherapy; CT, chemotherapy.

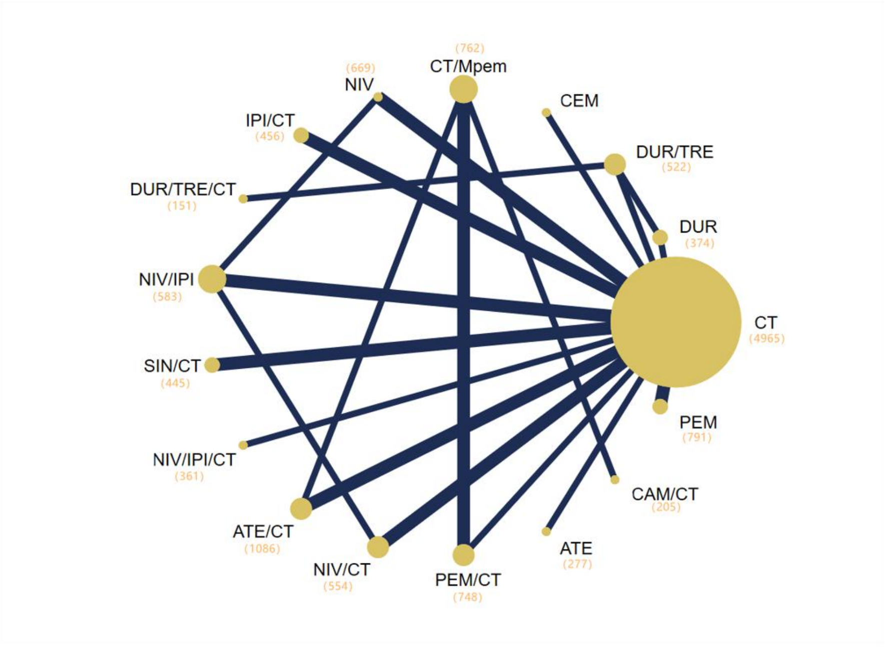

Supplementary Figure 15. Network diagram of comparisons on overall survival of specific treatment regimens. Each circular node represents a type of treatment. Each line represents a type of head-to-head comparison. The size of the nodes and the thickness of the lines are weighted according to the number of studies evaluating each treatment and direct comparison, respectively. The total number of patients receiving a treatment was shown in brackets. PEM, pembrolizumab; CEM, cemiplimab; SIN, sintilimab; ATE, atezolizumab; NIV, nivolumab; DUR, durvalumab; TRE, tremelimumab; CAM, camrelizumab; IPI, ipilimumab; CT, chemotherapy; CT+Mpem, CT followed by maintenance of pemetrexed.

A

PFS

OS

**PEM**	1.30 (0.69, 2.35)	0.74 (0.44, 1.18)	1.00 (0.53, 1.79)	0.88 (0.43, 1.74)	0.62 (0.33, 1.10)	0.92 (0.40, 2.00)	0.97 (0.58, 1.56)	1.14 (0.67, 1.82)	1.13 (0.60, 2.02)	1.33 (0.75, 2.22)	1.51 (0.88, 2.46)	1.08 (0.66, 1.71)	0.96 (0.57, 1.58)	1.08 (0.48, 2.29)	0.66 (0.37, 1.13)	0.77 (0.53, 1.07)
1.14 (0.86, 1.50)	**CEM**	0.56 (0.31, 1.03)	0.77 (0.38, 1.55)	0.68 (0.31, 1.48)	**0.47 (0.23, 0.95)**	0.70 (0.29, 1.69)	0.75 (0.41, 1.37)	0.87 (0.47, 1.60)	0.87 (0.43, 1.75)	1.02 (0.53, 1.93)	1.16 (0.62, 2.16)	0.83 (0.46, 1.51)	0.74 (0.40, 1.39)	0.83 (0.35, 1.94)	**0.50 (0.26, 0.98)**	**0.59 (0.36, 0.97)**
0.84 (0.70, 1.01)	**0.74 (0.56, 0.98)**	**NIV**	1.35 (0.74, 2.48)	1.20 (0.60, 2.40)	0.84 (0.46, 1.52)	1.25 (0.56, 2.77)	1.32 (0.87, 2.01)	1.55 (0.95, 2.48)	1.54 (0.84, 2.80)	**1.80 (1.05, 3.06)**	**2.05 (1.23, 3.40)**	1.47 (0.92, 2.35)	1.31 (0.79, 2.19)	1.46 (0.68, 3.17)	0.89 (0.51, 1.56)	1.04 (0.74, 1.47)
0.93 (0.71, 1.23)	0.82 (0.58, 1.16)	1.11 (0.83, 1.47)	**ATE**	0.89 (0.40, 1.93)	0.62 (0.31, 1.25)	0.92 (0.38, 2.23)	0.98 (0.53, 1.79)	1.14 (0.61, 2.09)	1.13 (0.56, 2.29)	1.33 (0.69, 2.54)	1.51 (0.81, 2.83)	1.09 (0.60, 1.98)	0.96 (0.52, 1.82)	1.08 (0.46, 2.55)	0.66 (0.34, 1.29)	0.77 (0.47, 1.27)
**0.81 (0.66, 0.99)**	**0.71 (0.53, 0.95)**	0.96 (0.77, 1.19)	0.87 (0.64, 1.17)	**DUR**	0.70 (0.37, 1.30)	1.04 (0.46, 2.35)	1.10 (0.55, 2.20)	1.29 (0.64, 2.57)	1.28 (0.59, 2.78)	1.50 (0.72, 3.10)	1.71 (0.84, 3.46)	1.23 (0.62, 2.43)	1.09 (0.54, 2.23)	1.22 (0.49, 3.05)	0.74 (0.36, 1.57)	0.87 (0.48, 1.59)
0.82 (0.67, 1.01)	**0.72 (0.54, 0.97)**	0.98 (0.79, 1.21)	0.88 (0.66, 1.19)	1.02 (0.81, 1.29)	**DUR+TRE**	1.49 (0.88, 2.53)	1.58 (0.87, 2.88)	**1.85 (1.00, 3.36)**	1.84 (0.91, 3.68)	**2.15 (1.13, 4.07)**	**2.45 (1.32, 4.53)**	1.76 (0.98, 3.17)	1.56 (0.85, 2.93)	1.75 (0.75, 4.09)	1.07 (0.55, 2.07)	1.25 (0.76, 2.04)
0.93 (0.66, 1.32)	0.82 (0.55, 1.23)	1.11 (0.79, 1.57)	1.01 (0.67, 1.51)	1.16 (0.81, 1.66)	1.14 (0.86, 1.50)	**DUR+TRE+CT**	1.06 (0.48, 2.36)	1.24 (0.55, 2.74)	1.23 (0.52, 2.95)	1.44 (0.63, 3.30)	1.64 (0.73, 3.70)	1.18 (0.54, 2.61)	1.05 (0.47, 2.38)	1.17 (0.43, 3.19)	0.72 (0.31, 1.67)	0.84 (0.41, 1.72)
1.05 (0.88, 1.26)	0.92 (0.70, 1.22)	**1.25 (1.03, 1.51)**	1.13 (0.85, 1.50)	**1.30 (1.05, 1.62)**	**1.28 (1.03, 1.58)**	1.12 (0.79, 1.59)	**NIV+IPI**	1.17 (0.75, 1.80)	1.16 (0.64, 2.11)	1.36 (0.79, 2.31)	1.55 (0.93, 2.56)	1.11 (0.70, 1.77)	0.99 (0.60, 1.65)	1.11 (0.51, 2.39)	0.68 (0.39, 1.18)	0.79 (0.56, 1.11)
0.97 (0.80, 1.17)	0.85 (0.64, 1.13)	1.15 (0.95, 1.41)	1.04 (0.78, 1.39)	1.20 (0.97, 1.50)	1.18 (0.95, 1.47)	1.04 (0.73, 1.47)	0.92 (0.76, 1.12)	**NIV+CT**	0.99 (0.55, 1.83)	1.16 (0.68, 2.01)	1.32 (0.80, 2.23)	0.95 (0.60, 1.54)	0.84 (0.52, 1.43)	0.95 (0.44, 2.08)	0.58 (0.33, 1.02)	**0.67 (0.48, 0.97)**
1.17 (0.94, 1.47)	1.03 (0.76, 1.41)	**1.40 (1.11, 1.76)**	1.26 (0.92, 1.72)	**1.45 (1.13, 1.87)**	**1.43 (1.11, 1.83)**	1.25 (0.86, 1.82)	1.12 (0.89, 1.41)	1.21 (0.95, 1.53)	**NIV+IPI+CT**	1.17 (0.62, 2.22)	1.33 (0.72, 2.48)	0.96 (0.53, 1.73)	0.85 (0.46, 1.60)	0.95 (0.41, 2.23)	0.58 (0.30, 1.13)	0.68 (0.42, 1.11)
1.15 (0.93, 1.43)	1.01 (0.75, 1.37)	**1.37 (1.10, 1.71)**	1.24 (0.91, 1.68)	**1.43 (1.12, 1.82)**	**1.40 (1.10, 1.78)**	1.23 (0.86, 1.78)	1.10 (0.88, 1.37)	1.19 (0.95, 1.49)	0.98 (0.76, 1.27)	**PEM+CT**	1.14 (0.65, 1.99)	0.82 (0.53, 1.27)	0.72 (0.42, 1.28)	0.81 (0.43, 1.55)	**0.50 (0.35, 0.71)**	**0.58 (0.39, 0.88)**
1.31 (0.94, 1.83)	1.15 (0.77, 1.72)	**1.56 (1.11, 2.20)**	1.41 (0.94, 2.10)	**1.63 (1.14, 2.32)**	**1.59 (1.12, 2.27)**	1.40 (0.90, 2.19)	1.25 (0.89, 1.75)	1.35 (0.96, 1.91)	1.12 (0.77, 1.61)	1.14 (0.79, 1.63)	**SIN+CT**	0.72 (0.44, 1.18)	0.64 (0.38, 1.09)	0.72 (0.33, 1.57)	**0.44 (0.25, 0.78)**	**0.51 (0.35, 0.74)**
0.90 (0.75, 1.07)	0.79 (0.60, 1.04)	1.07 (0.89, 1.29)	0.96 (0.73, 1.28)	1.11 (0.90, 1.37)	1.09 (0.88, 1.34)	0.96 (0.68, 1.35)	0.85 (0.71, 1.03)	0.93 (0.76, 1.12)	**0.76 (0.61, 0.96)**	**0.78 (0.64, 0.94)**	**0.68 (0.49, 0.96)**	**ATE+CT**	0.89 (0.55, 1.47)	0.99 (0.51, 1.95)	**0.61 (0.40, 0.91)**	**0.71 (0.51, 0.98)**
0.86 (0.70, 1.04)	**0.75 (0.56, 1.00)**	1.02 (0.83, 1.25)	0.92 (0.69, 1.23)	1.06 (0.85, 1.33)	1.04 (0.83, 1.30)	0.92 (0.64, 1.30)	**0.81 (0.66, 1.00)**	0.88 (0.72, 1.09)	**0.73 (0.57, 0.93)**	**0.74 (0.59, 0.94)**	**0.65 (0.46, 0.93)**	0.95 (0.78, 1.17)	**IPI+CT**	1.12 (0.51, 2.44)	0.68 (0.38, 1.20)	0.80 (0.55, 1.15)
0.94 (0.63, 1.41)	0.83 (0.52, 1.31)	1.12 (0.75, 1.68)	1.01 (0.64, 1.60)	1.17 (0.77, 1.77)	1.14 (0.75, 1.74)	1.01 (0.61, 1.66)	0.90 (0.60, 1.35)	0.97 (0.64, 1.47)	0.80 (0.52, 1.23)	0.82 (0.56, 1.18)	0.72 (0.44, 1.18)	1.05 (0.72, 1.54)	1.10 (0.73, 1.66)	**CAM+CT**	0.61 (0.36, 1.04)	0.71 (0.36, 1.42)
**0.68 (0.54, 0.85)**	**0.60 (0.43, 0.82)**	0.81 (0.64, 1.02)	**0.73 (0.53, 1.00)**	0.84 (0.65, 1.09)	0.82 (0.64, 1.06)	0.72 (0.50, 1.06)	**0.64 (0.51, 0.82)**	**0.70 (0.55, 0.89)**	**0.58 (0.44, 0.76)**	**0.59 (0.50, 0.69)**	**0.52 (0.36, 0.75)**	**0.76 (0.63, 0.91)**	0.79 (0.62, 1.01)	**0.72 (0.52, 1.00)**	**CT+Mpem**	1.17 (0.75, 1.81)
**0.77 (0.69, 0.87)**	**0.68 (0.53, 0.87)**	0.92 (0.80, 1.05)	0.83 (0.65, 1.07)	0.96 (0.81, 1.13)	0.94 (0.80, 1.11)	0.83 (0.60, 1.14)	**0.74 (0.64, 0.84)**	**0.80 (0.69, 0.92)**	**0.66 (0.55, 0.80)**	**0.67 (0.56, 0.80)**	**0.59 (0.43, 0.81)**	**0.86 (0.76, 0.98)**	0.90 (0.78, 1.05)	0.82 (0.56, 1.21)	1.14 (0.94, 1.39)	**CT**

B

≥ 3AEs

ORR

**PEM**	1.31 (0.85, 2.03)	1.12 (0.80, 1.57)	**1.77 (1.11, 2.88)**	0.97 (0.63, 1.49)	**0.57 (0.38, 0.85)**	0.47 (0.21, 1.03)	**0.43 (0.31, 0.60)**	**0.18 (0.13, 0.25)**	**0.23 (0.16, 0.33)**	**0.26 (0.18, 0.39)**	**0.28 (0.18, 0.42)**	**0.19 (0.14, 0.26)**	**0.17 (0.12, 0.24)**	**0.18 (0.10, 0.32)**	**0.36 (0.24, 0.53)**	**0.33 (0.26, 0.41)**
0.60 (0.21, 1.77)	**CEM**	0.86 (0.54, 1.34)	1.35 (0.78, 2.38)	0.74 (0.44, 1.25)	**0.44 (0.27, 0.71)**	**0.36 (0.15, 0.83)**	**0.33 (0.21, 0.51)**	**0.14 (0.09, 0.21)**	**0.18 (0.11, 0.28)**	**0.20 (0.12, 0.33)**	**0.21 (0.13, 0.35)**	**0.14 (0.09, 0.22)**	**0.13 (0.08, 0.20)**	**0.14 (0.07, 0.26)**	**0.27 (0.17, 0.44)**	**0.25 (0.17, 0.36)**
1.10 (0.47, 2.61)	1.85 (0.62, 5.18)	**NIV**	1.58 (0.98, 2.60)	0.86 (0.56, 1.34)	**0.51 (0.34, 0.77)**	**0.42 (0.19, 0.93)**	**0.39 (0.29, 0.51)**	**0.16 (0.12, 0.22)**	**0.20 (0.14, 0.30)**	**0.24 (0.16, 0.35)**	**0.25 (0.16, 0.38)**	**0.17 (0.12, 0.23)**	**0.15 (0.10, 0.21)**	**0.16 (0.09, 0.28)**	**0.32 (0.22, 0.48)**	**0.30 (0.23, 0.38)**
1.50 (0.53, 4.48)	2.50 (0.73, 8.61)	1.36 (0.48, 4.04)	**ATE**	**0.55 (0.31, 0.95)**	**0.32 (0.19, 0.55)**	**0.27 (0.11, 0.63)**	**0.24 (0.15, 0.39)**	**0.10 (0.06, 0.16)**	**0.13 (0.08, 0.22)**	**0.15 (0.09, 0.25)**	**0.16 (0.09, 0.27)**	**0.11 (0.07, 0.17)**	**0.09 (0.06, 0.15)**	**0.10 (0.05, 0.20)**	**0.20 (0.12, 0.34)**	**0.19 (0.12, 0.28)**
1.45 (0.50, 4.49)	2.42 (0.70, 8.54)	1.32 (0.45, 4.02)	0.97 (0.28, 3.44)	**DUR**	**0.59 (0.40, 0.85)**	0.49 (0.22, 1.06)	**0.45 (0.29, 0.69)**	**0.19 (0.12, 0.29)**	**0.24 (0.15, 0.38)**	**0.27 (0.17, 0.44)**	**0.29 (0.17, 0.47)**	**0.20 (0.13, 0.30)**	**0.17 (0.11, 0.27)**	**0.19 (0.10, 0.35)**	**0.37 (0.23, 0.60)**	**0.34 (0.24, 0.49)**
1.54 (0.52, 4.70)	2.56 (0.73, 8.95)	1.39 (0.47, 4.17)	1.02 (0.29, 3.63)	1.06 (0.42, 2.64)	**DUR+TRE**	0.83 (0.42, 1.63)	0.76 (0.51, 1.13)	**0.32 (0.21, 0.47)**	**0.40 (0.26, 0.63)**	**0.46 (0.30, 0.73)**	**0.49 (0.30, 0.79)**	**0.33 (0.22, 0.49)**	**0.29 (0.19, 0.45)**	**0.32 (0.17, 0.58)**	**0.63 (0.40, 0.99)**	**0.58 (0.42, 0.80)**
0.81 (0.19, 3.57)	1.34 (0.27, 6.52)	0.73 (0.17, 3.21)	0.54 (0.11, 2.61)	0.55 (0.14, 2.10)	0.53 (0.19, 1.41)	**DUR+TRE+CT**	**0.92 (0.42, 2.04)**	**0.38 (0.17, 0.85)**	0.49 (0.22, 1.10)	0.56 (0.25, 1.28)	0.59 (0.26, 1.37)	**0.40 (0.18, 0.89)**	**0.35 (0.16, 0.80)**	**0.38 (0.15, 0.96)**	0.76 (0.34, 1.74)	0.70 (0.33, 1.51)
0.92 (0.41, 2.22)	1.55 (0.54, 4.39)	0.84 (0.43, 1.73)	0.62 (0.21, 1.76)	0.64 (0.22, 1.87)	0.60 (0.21, 1.77)	1.15 (0.27, 4.94)	**NIV+IPI**	**0.42 (0.31, 0.56)**	**0.53 (0.36, 0.77)**	**0.61 (0.42, 0.89)**	**0.64 (0.42, 0.97)**	**0.44 (0.32, 0.60)**	**0.39 (0.27, 0.55)**	**0.42 (0.23, 0.73)**	0.83 (0.56, 1.23)	**0.76 (0.61, 0.96)**
0.57 (0.25, 1.40)	0.96 (0.34, 2.79)	0.52 (0.24, 1.20)	0.38 (0.13, 1.11)	0.40 (0.13, 1.17)	0.37 (0.13, 1.12)	0.71 (0.17, 3.11)	0.62 (0.31, 1.28)	**NIV+CT**	1.28 (0.87, 1.87)	**1.47 (1.00, 2.15)**	**1.54 (1.02, 2.35)**	1.05 (0.76, 1.45)	0.93 (0.65, 1.33)	1.00 (0.57, 1.75)	**1.99 (1.35, 2.96)**	**1.84 (1.45, 2.32)**
0.71 (0.25, 2.10)	1.18 (0.35, 4.00)	0.64 (0.23, 1.89)	0.47 (0.14, 1.62)	0.49 (0.14, 1.71)	0.46 (0.13, 1.63)	0.88 (0.18, 4.24)	0.77 (0.27, 2.17)	1.23 (0.43, 3.51)	**NIV+IPI+CT**	1.15 (0.75, 1.77)	1.21 (0.77, 1.91)	0.82 (0.57, 1.19)	0.73 (0.49, 1.09)	0.78 (0.43, 1.42)	**1.56 (1.01, 2.41)**	**1.44 (1.07, 1.95)**
0.46 (0.19, 1.23)	0.78 (0.25, 2.42)	0.42 (0.17, 1.10)	**0.31 (0.10, 0.96)**	0.32 (0.10, 1.02)	**0.30 (0.10, 0.97)**	0.58 (0.13, 2.64)	0.50 (0.20, 1.27)	0.81 (0.32, 2.03)	0.66 (0.22, 2.01)	**PEM+CT**	1.05 (0.67, 1.67)	**0.72 (0.52, 0.99)**	**0.63 (0.42, 0.95)**	0.68 (0.41, 1.11)	**1.36 (1.02, 1.80)**	1.25 (0.92, 1.70)
0.69 (0.29, 1.71)	1.15 (0.39, 3.38)	0.62 (0.26, 1.54)	0.46 (0.15, 1.37)	0.47 (0.16, 1.44)	0.45 (0.15, 1.36)	0.85 (0.19, 3.80)	0.74 (0.31, 1.76)	1.20 (0.49, 2.85)	0.97 (0.33, 2.85)	1.48 (0.55, 3.79)	**SIN+CT**	0.68 (0.45, 1.02)	**0.6 (0.39, 0.93)**	0.65 (0.35, 1.20)	1.29 (0.81, 2.05)	1.19 (0.84, 1.67)
0.80 (0.35, 1.88)	1.34 (0.47, 3.76)	0.72 (0.33, 1.68)	0.53 (0.19, 1.50)	0.55 (0.19, 1.61)	0.52 (0.18, 1.52)	0.99 (0.24, 4.23)	0.86 (0.39, 1.94)	1.39 (0.61, 3.13)	1.13 (0.40, 3.11)	1.72 (0.80, 3.66)	1.16 (0.50, 2.72)	**ATE+CT**	0.89 (0.62, 1.25)	0.95 (0.58, 1.56)	**1.90 (1.42, 2.52)**	**1.74 (1.40, 2.17)**
1.05 (0.39, 2.45)	1.76 (0.53, 4.84)	0.96 (0.36, 2.21)	0.70 (0.21, 1.95)	0.73 (0.21, 2.06)	0.69 (0.20, 1.98)	1.30 (0.27, 5.48)	1.14 (0.42, 2.56)	1.84 (0.66, 4.12)	1.50 (0.44, 4.04)	2.27 (0.76, 5.57)	1.54 (0.54, 3.65)	1.32 (0.50, 2.91)	**IPI+CT**	1.07 (0.60, 1.91)	**2.14 (1.42, 3.25)**	**1.97 (1.51, 2.59)**
0.68 (0.18, 2.61)	1.13 (0.26, 4.84)	0.61 (0.17, 2.32)	0.45 (0.10, 1.93)	0.47 (0.11, 2.02)	0.44 (0.10, 1.94)	0.84 (0.14, 4.96)	0.73 (0.20, 2.70)	1.18 (0.31, 4.30)	0.96 (0.22, 4.02)	1.46 (0.49, 4.29)	0.99 (0.26, 3.78)	0.85 (0.27, 2.62)	0.64 (0.18, 2.74)	**CAM+CT**	**2.00 (1.33, 3.00)**	**1.84 (1.1, 3.09)**
1.59 (0.61, 4.28)	2.65 (0.84, 8.49)	1.44 (0.56, 3.87)	1.06 (0.33, 3.37)	1.09 (0.33, 3.57)	1.03 (0.32, 3.40)	1.97 (0.43, 9.16)	1.71 (0.66, 4.46)	**2.76 (1.05, 7.21)**	2.25 (0.70, 7.02)	**3.41 (1.85, 6.29)**	2.31 (0.85, 6.26)	1.99 (0.97, 4.01)	1.50 (0.59, 4.59)	2.34 (0.97, 5.66)	**CT+Mpem**	0.92 (0.67, 1.26)
1.33 (0.74, 2.53)	2.22 (0.94, 5.28)	1.21 (0.68, 2.24)	0.89 (0.37, 2.12)	0.92 (0.37, 2.27)	0.87 (0.35, 2.16)	1.65 (0.43, 6.32)	1.44 (0.81, 2.57)	**2.32 (1.27, 4.19)**	1.88 (0.79, 4.47)	**2.87 (1.39, 5.84)**	**1.94 (1.02, 3.70)**	1.66 (0.96, 2.93)	1.26 (0.70, 2.77)	1.96 (0.61, 6.37)	0.84 (0.39, 1.80)	**CT**

Supplementary Figure 16. Network meta-analysis composed of specific treatment regimens. (A) Pooled hazard ratio (HR) (95% CrIs (credible intervals)) for overall survival (OS) and progression-free survival (PFS) in comparisons of each specific treatment regimen. (B) Pooled odds ratio (OR) (95% CrIs) for objective response rate (ORR) and adverse events of grade 3 or higher (≥3AEs) in comparisons of each specific treatment regimen. Data in each cell are HR or OR (95% CrIs) for the comparison of upper row-defining treatment versus lower row-defining treatment. HR less than 1 and OR more than 1 favour upper-row treatment. Significant results are highlighted in red and bold. PEM, pembrolizumab; CEM, cemiplimab; SIN, sintilimab; ATE, atezolizumab; NIV, nivolumab; DUR, durvalumab; TRE, tremelimumab; CAM, camrelizumab; IPI, ipilimumab; CT, chemotherapy; CT+Mpem, CT followed by maintenance of pemetrexed.

2 Supplementary Tables

Supplementary Table 1. Checklist of the PRISMA extension for network meta-analysis.

Section/topic	Item#	Checklist item*	Reported on page #
**TITLE**			
Title	1	Identify the report as a systematic review *incorporating a network meta- analysis (or related form of meta-analysis).*	1
**ABSTRACT**			
Structured summary	2	Provide a structured summary including, as applicable: • Background: main objectives; • Methods: data sources; study eligibility criteria, participants, and interventions; study appraisal and *synthesis methods, such as network meta- analysis.* • Results: number of studies and participants identified; summary estimates with corresponding confidence/credible intervals; *treatment rankings may also be discussed. Authors may choose to summarize pairwise comparisons against a chosen treatment included in their analyses for brevity.* • Discussion/Conclusions: limitations; conclusions and implicationsof findings. • Other: primary source of funding; systematic review registration number with registry name.	1-2
**INTRODUCTION**			
Rationale	3	Describe the rationale for the review in the context of what is already known, *including mention of why a network meta-analysis has been conducted.*	2
Objectives	4	Provide an explicit statement of questions being addressed with reference to participants, interventions, comparisons, outcomes, and study design (PICOS).	2
**METHODS**			
Protocol and registration	5	Indicate if a review protocol exists, if and where it can be accessed (e.g., Web address), and, if available, provide registration information including registration number.	2-3
Eligibility criteria	6	Specify study characteristics (e.g., PICOS, length of follow-up) and report characteristics (e.g., years considered, language, publication status) used as criteria for eligibility, giving rationale. *Clearly describe eligible treatments included in the treatment network, and note whether any have been clustered or merged into the same node (with justification).*	3
Information sources	7	Describe all information sources (e.g., databases with dates of coverage, contact with study authors to identify additional studies) in the search and date last searched.	2-3
Search	8	Present full electronic search strategy for at least one database, including any limits used, such that it could be repeated.	Supplementary Table 2
Study selection	9	State the process for selecting studies (i.e., screening, eligibility, included in systematic review, and, if applicable, included in the meta-analysis).	Figure 1
Data collection process	10	Describe method of data extraction from reports (e.g., piloted forms, independently, in duplicate) and any processes for obtaining and confirming data from investigators.	3
Data items	11	List and define all variables for which data were sought (e.g., PICOS, funding sources) and any assumptions and simplifications made.	3
Geometry of the network	S1	Describe methods used to explore the geometry of the treatment network under study and potential biases related to it. This should include how the evidence base has been graphically summarized for presentation, and what characteristics were compiled and used to describe the evidence base to readers.	3
Risk of bias in individual studies	12	Describe methods used for assessing risk of bias of individual studies (including specification of whether this was done at the study or outcome level), and how this information is to be used in any data synthesis.	3
Summary measures	13	State the principal summary measures (e.g., risk ratio, difference in means). *Also describe the use of additional summary measures assessed, such as treatment rankings and surface under the cumulative ranking curve (SUCRA) values, as well as modified approaches used to present summary findings from meta-analyses.*	3

Synthesis of results	14	Describe the methods of handling data and combining results of studies for each network meta-analysis. This should include, but not be limited to:<br>• *Handling of multi-arm trials;*<br>• *Selection of variance structure;*<br>• *Selection of prior distributions in Bayesian analyses; and*<br>• *Assessment of model fit.*	3
Assessment of Inconsistency	S2	Describe the statistical methods used to evaluate the agreement of direct and indirect evidence in the treatment network(s) studied. Describe efforts taken to address its presence when found.	3
Risk of bias across studies	15	Specify any assessment of risk of bias that may affect the cumulative evidence (e.g., publication bias, selective reporting within studies).	3
Additional analyses	16	Describe methods of additional analyses, if done, indicating which were pre- specified. This may include, but not be limited to the following:<br>• Sensitivity or subgroup analyses;<br>• Meta-regression analyses;<br>• *Alternative formulations of the treatment network; and*<br>• *Use of alternative prior distributions for Bayesian analyses (if applicable).*	3
**RESULTS**			
Study selection	17	Give numbers of studies screened, assessed for eligibility, and included in the review, with reasons for exclusions at each stage, ideally with a flow diagram.	3
Presentation of network structure	S3	Provide a network graph of the included studies to enable visualization of the geometry of the treatment network	6
Summary of network geometry	S4	Provide a brief overview of characteristics of the treatment network. This may include commentary on the abundance of trials and randomized patients for the different interventions and pairwise comparisons in the network, gaps of evidence in the treatment network, and potential biases reflected by the network structure.	3
Study characteristics	18	For each study, present characteristics for which data were extracted (e.g., study size, PICOS, follow-up period) and provide the citations.	3
Risk of bias within studies	19	Present data on risk of bias of each study and, if available, any outcome level assessment.	3
Results of individual studies	20	For all outcomes considered (benefits or harms), present, for each study:1) simple summary data for each intervention group, and 2) effect estimates and confidence/credible intervals. *Modified approaches may be needed to deal with information from larger networks.*	3-9
Synthesis of results	21	Present results of each meta-analysis done, including confidence/credible intervals. *In larger networks, authors may focus on comparisons versus a particular comparator (e.g. placebo or standard care), with full findings presented in an appendix. League tables and forest plots may be considered to summarize pairwise comparisons.* If additional summary measures were explored (such as treatment rankings), these should also be presented.	3-9
Exploration for inconsistency	S5	Describe results from investigations of inconsistency. This may include such information as measures of model fit to compare consistency and inconsistency models, *P* values from statistical tests, or summary of inconsistency estimates from different parts of the treatment network.	8-9
Risk of bias across studies	22	Present results of any assessment of risk of bias across studies.	3
Additional analysis	23	Give results of additional analyses, if done (e.g., sensitivity or subgroup analyses, meta-regression, *alternative network geometries studied, alternative choice of prior distributions for Bayesian analyses,* and so forth.	4-9

**DISCUSSION**			
Summary of evidence	24	Summarize the main findings including the strength of evidence for each main outcome; consider their relevance to key groups (e.g., healthcare providers, users, and policy makers).	9-10
Limitations	25	Discuss limitations at study and outcome level (e.g., risk of bias), and at review-level (e.g., incomplete retrieval of identified research, reporting bias). *Comment on the validity of the assumptions, such as transitivity and consistency. Comment on any concerns regarding network geometry (e.g., avoidance of certain comparisons).*	10
Conclusions	26	Provide a general interpretation of the results in the context of other evidence, and implications for future research.	10
FUNDING			
Funding	27	Describe sources of funding for the systematic review and other support (e.g., supply of data); role of funders for the systematic review.	10

PICOS: population, intervention, comparators, outcomes, study design.
*Text in italics indicates wording specific to reporting of network meta-analyses that has been added to guidance from the PRISMA statement.
† Authors may wish to plan for use of appendices to present all relevant information in full detail for items in this section.

Supplementary Table 2. Literature search criteria.
(A) Search strategy on Pubmed.

(((((((((Carcinoma, Non-Small-Cell Lung[MeSH Terms]) OR (NSCLC[Title/Abstract])) OR ("Non Small Cell"[Title/Abstract])) OR ("Non-Small-Cell"[Title/Abstract])) OR ("Non-Small Cell"[Title/Abstract])) AND (((((((((((((((((((((((((((((((((((((("programmed death 1"[Title/Abstract]) OR (PD-1[Title/Abstract])) OR (PD1[Title/Abstract])) OR ("programmed death ligand 1"[Title/Abstract])) OR (PD-L1[Title/Abstract])) OR (PDL1[Title/Abstract])) OR ("PD L1"[Title/Abstract])) OR ("PD 1"[Title/Abstract])) OR (anti-PD-1[Title/Abstract])) OR (anti-PD-L1[Title/Abstract])) OR (Immunotherapy[MeSH Terms])) OR (Immunotherap*[Title/Abstract])) OR ("immune checkpoint"[Title/Abstract])) OR ((ICB[Title/Abstract])) OR ("CTLA-4"[Title/Abstract])) OR ("CTLA 4"[Title/Abstract])) OR (CTLA4[Title/Abstract])) OR (LAG-3[Title/Abstract])) OR (LAG3[Title/Abstract])) OR ("LAG 3"[Title/Abstract])) OR (TIM-3[Title/Abstract])) OR (TIM3[Title/Abstract])) OR ("TIM 3"[Title/Abstract])) OR (TIGIT[Title/Abstract])) OR (VISTA[Title/Abstract])) OR (Tislelizumab[Title/Abstract])) OR (toripalimab[Title/Abstract])) OR (camrelizumab[Title/Abstract])) OR (sintilimab[Title/Abstract])) OR (avelumab[Title/Abstract])) OR (durvalumab[Title/Abstract])) OR (atezolizumab[Title/Abstract])) OR (Nivolumab[Title/Abstract])) OR (pembrolizumab[Title/Abstract])) OR (BMS936559[Title/Abstract])) OR (Pidilizumab[Title/Abstract])) OR (Ipilimumab[Title/Abstract])) OR (Tremelimumab[Title/Abstract]))) AND (((((((((Randomized Controlled Trial[Publication Type]) OR (controlled clinical trial[Publication Type])) OR ("Randomized Controlled Trial"[Title/Abstract])) OR ("controlled clinical trial"[Title/Abstract])) OR (randomized[Title/Abstract])) OR (randomised[Title/Abstract])) OR (randomly[Title/Abstract])) OR (trial[Title/Abstract])) OR (phase[Title/Abstract])) AND (((((first[Title/Abstract]) OR (1st[Title/Abstract])) OR (naive[Title/Abstract])) OR (naïve[Title/Abstract])) OR (untreated[Title/Abstract])))) AND (("2005"[Date - Publication] : "2020"[Date - Publication]))

(B) Search strategy on Embase.

('non small cell lung cancer'/exp OR nsclc:ab,ti OR 'non small cell':ab,ti OR 'non-small-cell':ab,ti OR 'non-small cell':ab,ti) AND ('immunotherapy'/exp OR immunotherap*:ab,ti OR 'immune checkpoint':ab,ti OR icb:ab,ti OR 'programmed death 1 receptor'/exp OR pd1:ab,ti OR 'pd 1':ab,ti OR 'programmed death 1':ab,ti OR 'programmed death 1 ligand 1'/exp OR pdl1:ab,ti OR 'pd l1':ab,ti OR 'programmed death 1 ligand 1':ab,ti OR 'anti pd 1':ab,ti OR 'anti pd l1':ab,ti OR 'cytotoxic t lymphocyte antigen 4'/exp OR 'ctla 4':ab,ti OR ctla4:ab,ti OR lag3:ab,ti OR 'lag 3':ab,ti OR tim3:ab,ti OR 'tim 3':ab,ti OR tigit:ab,ti OR vista:ab,ti OR tislelizumab:ab,ti OR toripalimab:ab,ti OR camrelizumab:ab,ti OR sintilimab:ab,ti OR avelumab:ab,ti OR durvalumab:ab,ti OR atezolizumab:ab,ti OR nivolumab:ab,ti OR pembrolizumab:ab,ti OR bms936559:ab,ti OR pidilizumab:ab,ti OR ipilimumab:ab,ti OR tremelimumab:ab,ti) AND (first:ab,ti OR 1st:ab,ti OR front:ab,ti OR naive:ab,ti OR naïve:ab,ti OR untreated:ab,ti) AND [randomized controlled trial]/lim AND [2005-2020]/py

(C) Search strategy on Cochrane library.

#1 MeSH descriptor: [Carcinoma, Non-Small-Cell Lung] explode all trees
#2 MeSH descriptor: [Immunotherapy] explode all trees
#3 MeSH descriptor: [Programmed Cell Death 1 Receptor] explode all trees
#4 MeSH descriptor: [CTLA-4 Antigen] explode all trees
#5 (Immunotherap*):ti,ab,kw OR ("immune checkpoint"):ti,ab,kw OR (ICB):ti,ab,kw OR (pd1):ti,ab,kw OR ("pd 1"):ti,ab,kw (Word variations have been searched)
#6 (pd-1):ti,ab,kw OR ("programmed death 1"):ti,ab,kw OR (pdl1):ti,ab,kw OR ("pd l1"):ti,ab,kw OR (pd-l1):ti,ab,kw (Word variations have been searched)
#7 ("programmed death ligand 1"):ti,ab,kw OR (anti-PD-1):ti,ab,kw OR (anti-PD-L1):ti,ab,kw OR (CTLA-4):ti,ab,kw OR ("CTLA 4"):ti,ab,kw (Word variations have been searched)
#8 (CTLA4):ti,ab,kw OR (LAG-3):ti,ab,kw OR (LAG3):ti,ab,kw OR ("LAG 3"):ti,ab,kw OR (TIM-3):ti,ab,kw (Word variations have been searched)
#9 (TIM3):ti,ab,kw OR ("TIM 3"):ti,ab,kw OR (TIGIT):ti,ab,kw OR (VISTA):ti,ab,kw OR (Tislelizumab):ti,ab,kw (Word variations have been searched)
#10 (toripalimab):ti,ab,kw OR (Camrelizumab):ti,ab,kw OR (sintilimab):ti,ab,kw OR (avelumab):ti,ab,kw OR (durvalumab):ti,ab,kw
#11 (atezolizumab):ti,ab,kw OR (Nivolumab):ti,ab,kw OR (pembrolizumab):ti,ab,kw OR (BMS936559):ti,ab,kw OR (Pidilizumab):ti,ab,kw
#12 (Ipilimumab):ti,ab,kw OR (Tremelimumab):ti,ab,kw
#13 #2 OR #3 OR #4 OR #5 OR #6 OR #7 OR #8 OR #9 OR #10 OR #11 OR #12
#14 ("non small cell lung cancer"):ti,ab,kw OR (NSCLC):ti,ab,kw OR ("Non small cell"):ti,ab,kw OR (Non-small-cell):ti,ab,kw OR ("Non-small cell"):ti,ab,kw
#15 #1 OR #14
#16 (first):ti,ab,kw OR (1st):ti,ab,kw OR (naive):ti,ab,kw OR (naïve):ti,ab,kw OR (untreated):ti,ab,kw
#17 (front):ti,ab,kw
#18 #16 OR #17
#19 #13 AND #15 AND #18
with Cochrane Library publication date from Jan 2005 to Dec 2020, in Trials

Supplementary Table 3. Comparisons of the fit of consistency and inconsistency models using deviance information criteria (DIC).

		model		
		consistency,fixed	consistency,random	inconsistency
Overall	OS	**50.18**	**40.22**	**41.12**
	PFS	**116.39**	**45.23**	**45.56**
	ORR	**142.52**	**87.78**	**89.68**
	≥3AEs	72.82	71.69	74.72
Squamous	OS	22.86	22.99	24.73
	PFS	**36.81**	**20.73**	**20.71**
Non squamous	OS	**38.67**	**31.73**	**32.25**
	PFS	**42.38**	**24.45**	**24.46**
PD-L1＜1%	OS	15.00	16.33	15.00
	PFS	14.06	15.13	14.07
PD-L1≥1%	OS	25.69	24.21	25.70
	PFS	27.91	24.13	27.92
PD-L1 1-49%	OS	17.35	17.23	17.35
	PFS	14.62	13.28	14.62
PD-L1≥50%	OS	18.51	20.33	19.43
	PFS	**31.39**	**24.34**	**24.28**
High TMB	OS	13.43	14.39	13.43
	PFS	12.68	13.10	12.67
Low TMB	OS	17.03	15.22	17.04
	PFS	18.22	14.20	18.20
Ever smoking	OS	24.29	21.17	26.29
	PFS	11.05	11.25	11.05
Never smoking	OS	22.91	21.13	24.08
	PFS	6.08	7.80	6.08
Male	OS	12.96	14.69	14.92
	PFS	11.62	12.72	11.62
Female	OS	**36.80**	**21.65**	**21.72**
	PFS	11.06	11.96	11.07
Age≥65	OS	15.47	16.87	17.33
	PFS	7.19	9.01	7.20
Age<65	OS	28.34	23.28	29.87
	PFS	17.40	14.79	17.39
ECOG PS=0	OS	21.21	19.28	21.20
	PFS	8.00	9.64	8.02
ECOG PS=1	OS	19.40	19.63	19.40
	PFS	8.56	10.25	8.55
**The sensitive analysis by excluding trials with highly selected populations**				

**Overall**	**OS**	35.80	32.34	37.22
	**PFS**	**67.21**	**34.82**	**35.44**
	**ORR**	**110.10**	**69.96**	**70.46**
	**≥3AEs**	62.36	60.81	64.70
**Squamous**	**OS**	17.61	17.92	19.23
	**PFS**	**27.74**	**13.06**	**13.05**
**Non squamous**	**OS**	26.28	23.28	28.19
	**PFS**	15.28	14.86	15.27

Number highlighted in bold indicates DIC value was higher using fixed consistency model than inconsistency model, where random model was used.

OS, overall survival; PFS, progression-free survival; ORR, objective response rate; ≥3AEs, adverse events of grade 3 or higher; PD-L1, programmed-death ligand 1; TMB, tumor mutation burden; ECOG PS, Eastern Cooperative Oncology Group Performance Status.

Supplementary Table 4. Node-splitting analysis of inconsistency.

Nodes	Direct effect	Indirect effect	Overall	P
**Overall survival**				
DICI vs CT	0.79(0.64,0.97)	0.75(0.44,1.3)	0.77(0.65,0.91)	0.84
DICI+CT vs CT	0.66(0.48,0.91)	0.68(0.44,1.0)	0.67(0.52,0.86)	0.89
DICI vs SICI	0.94(0.70,1.3)	0.85(0.62,1.2)	0.94(0.78,1.1)	0.63
SICI+CT vs DICI	1.2(0.72,2.1)	0.92(0.74,1.1)	0.98(0.82,1,2)	0.32
DICI+CT vs DICI	0.88(0.60,1.3)	0.85(0.59,1.2)	0.86(0.67,1.1)	0.9
**Progression-free survival**				
DICI vs CT	0.92(0.68,1.3)	1.0(0.47,2.2)	0.89(0.69,1.1)	0.82
DICI+CT vs CT	0.68(0.41,1.1)	0.58(0.32,1.1)	0.64(0.43,0.93)	0.7
DICI vs SICI	1.0(0.65,1.6)	1.0(0.63,1.6)	1.1(0.81,1.5)	1
SICI+CT vs DICI	0.97(0.49,1.9)	0.64(0.45,0.9)	0.71(0.53,0.94)	0.27
DICI+CT vs DICI	0.67(0.39,1.2)	0.78(0.44,1.4)	0.72(0.49,1.1)	0.7
**Objective response rate**				
DICI vs SICI	1.2 (0.66, 2.2)	0.81 (0.38, 1.7)	0.99 (0.63, 1.5)	0.39
SICI+CT vs DICI	1.6 (0.66, 4.)	1.7 (0.96, 3.0)	1.7 (1.1, 2.6)	0.95
DICI+CT vs DICI	1.9 (0.73, 5.)	1.5 (0.58, 3.7)	1.7 (0.86, 3.2)	0.69
CT vs DICI	0.88 (0.53, 1.5)	1.0 (0.28, 3.7)	0.80 (0.53, 1.2)	0.84
CT vs DICI+CT	0.53 (0.24, 1.2)	0.41 (0.14, 1.2)	0.49 (0.26, 0.91)	0.69
**Grade ≥3 adverse events**				
DICI vs SICI	2.1 (1.6, 2.6)	2.6 (1.8, 3.8)	2.4 (2., 2.9)	0.31
SICI+CT vs DICI	3.4 (2.2, 5.4)	2.4 (1.9, 3.1)	2.6 (2.1, 3.2)	0.18
DICI+CT vs DICI	1.2 (0.61, 2.4)	2.1 (1.5, 3.)	1.9 (1.4, 2.6)	0.15
CT vs DICI	1.3 (1.1, 1.6)	0.84 (0.40, 1.8)	1.4 (1.2, 1.7)	0.24
CT vs DICI+CT	0.70 (0.52, 0.94)	1.2 (0.60, 2.5)	0.76 (0.57, 1.0)	0.15
**Overall survival for squamous**				
DICI+CT vs DICI	1.1 (0.65, 1.9)	0.98 (0.65, 1.5)	1.0 (0.74, 1.4)	0.72
CT vs DICI	1.6 (1.2, 2.0)	1.8 (0.96, 3.3)	1.6 (1.3, 2.0)	0.72
CT vs DICI+CT	1.6 (1.2, 2.2)	1.4 (0.80, 2.6)	1.6 (1.2, 2.1)	0.72
**Overall survival for non-squamous**				
DICI+CT vs DICI	0.76 (0.46, 1.3)	0.90 (0.51, 1.6)	0.82 (0.57, 1.2)	0.63
CT vs DICI	1.3 (0.94, 1.8)	1.1 (0.55, 2.2)	1.3 (0.96, 1.7)	0.62
CT vs DICI+CT	1.4 (0.91, 2.3)	1.7 (0.94, 3.2)	1.5 (1.1, 2.2)	0.63
**Overall survival for PD-L1<1%**				
SICI+CT vs DICI	1.2 (0.77, 1.9)	1.0 (0.71, 1.5)	1.1 (0.91, 1.4)	0.59
**Overall survival for PD-L1 1-49%**				
DICI vs SICI	1.2 (0.50, 2.9)	1.0 (0.76, 1.3)	1.0 (0.80, 1.3)	0.68
**Overall survival for PD-L1≥50%**				
DICI vs SICI	0.93 (0.63, 1.4)	1.5 (0.81, 2.9)	1.1 (0.86, 1.3)	0.19
DICI+CT vs DICI	0.64 (0.40, 1.0)	0.91 (0.58, 1.4)	0.77 (0.56, 1.1)	0.3

CT vs DICI	1.4 (1.1, 1.7)	0.97 (0.52, 1.8)	1.3 (1.1, 1.6)	0.29
CT vs DICI+CT	1.5 (1.0, 2.3)	2.2 (1.3, 3.6)	1.7 (1.3, 2.4)	0.29
**Overall survival for high TMB**				
DICI vs SICI	0.68 (0.31, 1.5)	0.98 (0.68, 1.4)	0.87 (0.64, 1.2)	0.41
**Progression-free survival for high TMB**				
DICI vs SICI	0.69 (0.30, 1.6)	0.94 (0.52, 1.7)	0.79 (0.53, 1.2)	0.55
**Overall survival for low TMB**				
DICI vs SICI	1.2 (0.80, 1.9)	0.71 (0.53, 0.95)	0.94 (0.76, 1.2)	**<u>0.04</u>**
**Progression-free survival for low TMB**				
DICI vs SICI	1.3 (0.82, 2.1)	0.59 (0.39, 0.89)	0.95 (0.74, 1.2)	**<u>0.01</u>**
**Overall survival for ever smoking**				
DICI+CT vs DICI	0.85 (0.64, 1.1)	0.86 (0.66, 1.1)	0.86 (0.71, 1.0)	0.94
CT vs DICI	1.4 (1.2, 1.6)	1.4 (0.96, 2.)	1.4 (1.2, 1.6)	0.94
CT vs DICI+CT	1.6 (1.3, 2.0)	1.6 (1.2, 2.2)	1.6 (1.4, 1.9)	0.94
**Overall survival for never smoking**				
DICI+CT vs DICI	0.72 (0.31, 1.7)	1.2 (0.60, 2.3)	0.97 (0.58, 1.6)	0.36
CT vs DICI	1.0 (0.71, 1.5)	0.63 (0.23, 1.7)	0.98 (0.69, 1.4)	0.36
CT vs DICI+CT	0.88 (0.50, 1.5)	1.4 (0.58, 3.6)	1.0 (0.62, 1.6)	0.36
**Overall survival for male**				
DICI+CT vs DICI	1.0 (0.72, 1.4)	0.97 (0.73, 1.3)	0.99 (0.79, 1.2)	0.86
CT vs DICI	1.5 (1.2, 1.7)	1.5 (1.0, 2.3)	1.5 (1.3, 1.7)	0.86
CT vs DICI+CT	1.5 (1.2, 1.9)	1.5 (1.0, 2.1)	1.5 (1.2, 1.8)	0.86
**Overall survival for female**				
DICI+CT vs DICI	0.67 (0.19, 2.3)	0.76 (0.14, 4.3)	0.70 (0.29, 1.7)	0.88
CT vs DICI	1.1 (0.34, 3.7)	0.99 (0.17, 5.7)	1.1 (0.45, 2.6)	0.88
CT vs DICI+CT	1.5 (0.43, 5.1)	1.7 (0.30, 9.5)	1.5 (0.63, 3.8)	0.89
**Overall survival for age≥65**				
DICI+CT vs DICI	0.85 (0.58, 1.2)	0.94 (0.66, 1.3)	0.90 (0.70, 1.2)	0.71
CT vs DICI	1.3 (1.1, 1.6)	1.2 (0.73, 1.9)	1.3 (1.1, 1.5)	0.71
CT vs DICI+CT	1.4 (1.0, 1.8)	1.5 (0.98, 2.3)	1.4 (1.1, 1.8)	0.71
**Overall survival for age<65**				
DICI+CT vs DICI	0.73 (0.50, 1.1)	0.87 (0.62, 1.2)	0.81 (0.63, 1.0)	0.5
CT vs DICI	1.4 (1.2, 1.7)	1.2 (0.74, 1.9)	1.4 (1.2, 1.7)	0.49
CT vs DICI+CT	1.6 (1.2, 2.2)	2. (1.3, 3.0)	1.7 (1.4, 2.2)	0.49

Significant values are in bold and underlined, indicating a significant inconsistency between the direct effect and indirect effect.

OS, overall survival; PFS, progression-free survival; ORR, objective response rate; ≥3AEs, adverse events of grade 3 or higher; PD-L1, programmed-death ligand 1; TMB, tumor mutation burden; ECOG PS, Eastern Cooperative Oncology Group Performance Status; SICI, Single immune checkpoint inhibitor; DICI, double immune checkpoint inhibitors; SICI+CT, Singlet immune checkpoint inhibitor combined with chemotherapy; DICI+CT, double immune checkpoint inhibitors combined with chemotherapy; CT, chemotherapy.
